# H3 Histone Tail Conformation within the Nucleosome and the Impact of K14 Acetylation Studied Using Enhanced Sampling Simulation

**DOI:** 10.1371/journal.pcbi.1004788

**Published:** 2016-03-11

**Authors:** Jinzen Ikebe, Shun Sakuraba, Hidetoshi Kono

**Affiliations:** 1 Molecular Modeling and Simulation Group, Japan Atomic Energy Agency, Kizugawa, Japan; 2 Graduate School of Frontier Sciences, The University of Tokyo, Kashiwa-shi, Japan; National Cancer Institute, United States of America and Tel Aviv University, Israel, UNITED STATES

## Abstract

Acetylation of lysine residues in histone tails is associated with gene transcription. Because histone tails are structurally flexible and intrinsically disordered, it is difficult to experimentally determine the tail conformations and the impact of acetylation. In this work, we performed simulations to sample H3 tail conformations with and without acetylation. The results show that irrespective of the presence or absence of the acetylation, the H3 tail remains in contact with the DNA and assumes an α-helix structure in some regions. Acetylation slightly weakened the interaction between the tail and DNA and enhanced α-helix formation, resulting in a more compact tail conformation. We inferred that this compaction induces unwrapping and exposure of the linker DNA, enabling DNA-binding proteins (e.g., transcription factors) to bind to their target sequences. In addition, our simulation also showed that acetylated lysine was more often exposed to the solvent, which is consistent with the fact that acetylation functions as a post-translational modification recognition site marker.

## Author Summary

Post-translational modification (PTM) of histone tails is an important component of epigenetics. Acetylation of histone tails generally functions to activate gene expression, though the molecular mechanism is not well understood. We used enhanced sampling simulation to examine the impact of acetylation on the structure of the histone H3 tail within the nucleosome. The results suggest acetylation makes the H3 tail conformation more compact and enhances dissociation of nucleosomal DNA from the histone core. Further, the acetylated lysine was more exposed to the solvent, which is consistent with its role as a PTM recognition site marker. These findings increase our understanding of the impact of PTM on nucleosome stability and dynamics and on the higher order structure of chromatin.

## Introduction

In eukaryotic cells, the genome is compactly stored within the nucleus as a complex with proteins. The basic structural unit of the complex is the nucleosome, which is composed of 146 or 147 base pairs of DNA wrapped around a histone octamer consisting of two copies each of histones H3, H4, H2A and H2B[1]. Posttranslational modification (PTM) of histones, including acetylation, methylation, phosphorylation and ubiquitination, has been studied since the early days of epigenetics. PTM occurs most often in the N-terminal regions (tails) of histones, and the precise locations of PTM are closely linked to specific DNA functions and biological events[2]. This prompted Strahi and Allis to propose the *histone code hypothesis*[3]: “multiple histone modifications, acting in a combinatorial or sequential fashion on one or multiple histone tails, specify unique downstream functions.” For example, acetylation of lysine residues on the H3 and H4 tails generally activate transcription[4, 5]. However, because the tails are intrinsically disordered with no static conformation, details of the molecular mechanism by which PTM exerts its effects remain unclear[6, 7], and there are no decisive clues as to which extent PTM affects the conformations of histone tails, nucleosomes or chromatin.

Histone tails play a key role in partial charge neutralization of nucleosomes and contribute to nucleosome aggregation. For example, Allan et al.[8] found that nucleosomes without histone tails do not aggregate, and Krajewski et al.[9] reported that the H3 and H4 tails were especially important for chromatin folding. Histone tails contain a large number of positively charged lysine and arginine residues, and thus preferentially contact DNA via electrostatic interactions. The positive charges partially neutralize the negative charges of nucleosomal DNA and reduce the electrostatic repulsive forces among nucleosomes, thereby mediating nucleosome aggregation. On the other hand, lysine acetylation neutralizes the positive charge on lysine residues, which weakens the interactions between the tails and the DNA. Lee et al.[10] suggested that acetylation leads to dissociation of the tails from the DNA and/or induces a change in the DNA configuration within the histone core, which allows transcription factor binding. However, the impact of acetylation on nucleosome structure or the higher order structure of chromatin is not yet known.

The difficulty in understanding how the conformation of nucleosomes or chromatin is changed through PTM is attributable to a lack of conformational information about histone tails. Although the crystal structures of nucleosomes have been solved, the conformations of the histone tails could not be determined because they are structurally flexible unless in complex with a specific protein. In addition, the existence of DNA strongly affects conformation of the tails, because positively charged histone tails favorably interact with negatively charged DNA. Conformational sampling for the histone tails should be investigated in the presence of nucleosomal DNA. Therefore, previous simulation studies using an enhanced sampling method in explicit water obtained conformational ensembles for histone tails isolated from the nucleosome[11]. These simulations showed that histone tails were not just flexible chains, but chains that had intrinsic conformational preferences. To the best of our knowledge, however, there are no simulation reports in which tail conformation was studied in the context of the nucleosome, and so the effects of histone tails on the structure and stability of nucleosomes remain unclear.

In the present study, we used computer simulations to ask how the H3 tail behaves within the nucleosome and how acetylation of H3 on K14 induces changes in the chromatin conformation. Because histone tails are intrinsically disordered and thus difficult to experimentally characterize, molecular dynamics (MD) simulation was used to investigate the flexible peptide. Using an enhanced sampling method, adaptive lambda square dynamics (ALSD)[12], we carried out conformational sampling of H3 histone tails, with and without acetylation at K14, and determined which conformation is preferred in the presence of nucleosomal DNA. In addition, we investigated how a single acetylation affects tail conformation and the solvent accessibility of the acetylated residue.

## Results

### ALSD samples various conformations of the H3 tail

**Fig 1:**
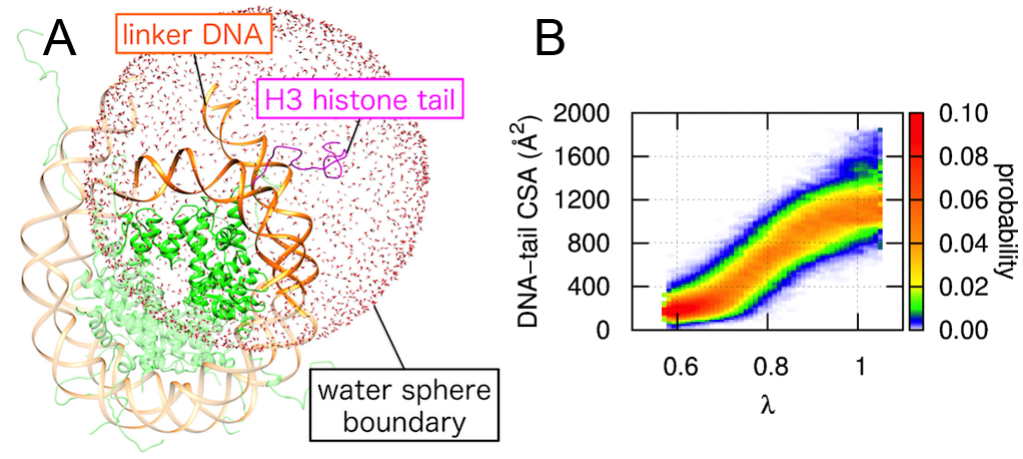
Simulation systems used and distribution of contact surface area (CSA) between the H3 tail and DNA in an obtained ensemble. (A) The system was prepared based on two nucleosome crystal structures, 1KX5 and 1ZBB. The linker DNA was extended by 10 bp from that of 1KX5 using the DNA structure of 1ZBB as a reference. Only atoms immersed within a water sphere centered at the root of the H3 tail (a nitrogen atom in the 40th residue) were considered in the simulations to reduce computational costs. All simulations were performed within the water sphere boundary. H3 histone tail, DNA, and histone core regions are shown in magenta, orange and green, respectively. (B) Distribution of CSA between the H3 tail and DNA is plotted as a function of the scaling factor λ for ALSD. See S1 Fig for the K14ac H3 tail.

In conventional MD simulations, the conformations of histone tails cannot be sufficiently sampled within a practical simulation time, because attractive electrostatic interactions strongly bind the tails to the DNA. We therefore applied ALSD enhanced sampling to obtain various conformations of the H3 tail within the context of a nucleosome (Fig 1A). We assessed the interaction of the H3 tail and the DNA based on the contact surface area (CSA) between the two. The CSA is the surface area of the DNA covered by the H3 tail and was computed as the difference between the solvent accessible surface area (SASA)[13] of the DNA, with and without the H3 tail. For the SASA calculation, atomic radii were set at 1.7 Å for carbon, 1.625 Å for nitrogen, 1.48 Å for oxygen, 1.87 Å for phosphate and 1.4 Å for water molecules. Hydrogen atoms were excluded from the calculation. In the ALSD simulation, a scaling factor, λ, was introduced to scale the potential energy of the H3 tail. As λ decreased, the potential energy was scaled down, and changes in the conformation of the tail were enhanced. Fig 1B and S1 Fig show the relation between the CSA distribution and λ in the unacetylated and K14-acetylated (K14ac) systems. Lower CSA values indicate dissociation of the tail from the DNA and elongation in the solvent. Our simulation results show that the CSA varied widely (0 to 1880 Å^2^) and declined with decreasing λ, demonstrating that ALSD sampled a broad range of H3 tail conformations. A sampled conformation at CSA = 0 is shown in S2 Fig.

### H3 tail is located in the vicinity of the DNA even after K14 acetylation

Ensembles reweighted to λ = 1 only give conformations seen under realistic conditions (corresponding to a temperature of 300 K) and have high CSA values. The average and standard error from 256 ALSD simulation runs with trivial trajectory parallelization (TTP)[14] were 1135 ± 10 and 1062 ± 9 Å^2^ for the unacetylated and K14ac system, respectively. These large CSA values indicate that the H3 tail is in the vicinity of the DNA, and does not adopt an elongated conformation (S2 Fig). The elongated dissociated H3 tail conformations are thermally unstable in the canonical ensemble at λ = 1, with and without K14 acetylation. Note, however, that a nucleosome model (PDB ID: 1KX5[15]) often used in the literature and textbooks has much lower CSA values (209 and 281 Å^2^ for chains A and E of the H3 histones) than those obtained in the present study, which indicates the tail is protruding from the nucleosome and might raise the possibility of giving an incorrect impression of the H3 tail conformation within the nucleosome. Our simulation results show that with or without K14 acetylation, H3 tails bind to the DNA, though K14 acetylation decreases the CSA slightly. We suggest that a conformation in which the H3 tail is dissociated from the nucleosomal DNA is not thermodynamically stable, even with K14 acetylation. Addition of acetylation at other sites on the H3 tail or other binding factors may be required to stabilize the dissociated state.

### K14ac increases the α-helical content of the H3 tail

**Fig 2:**
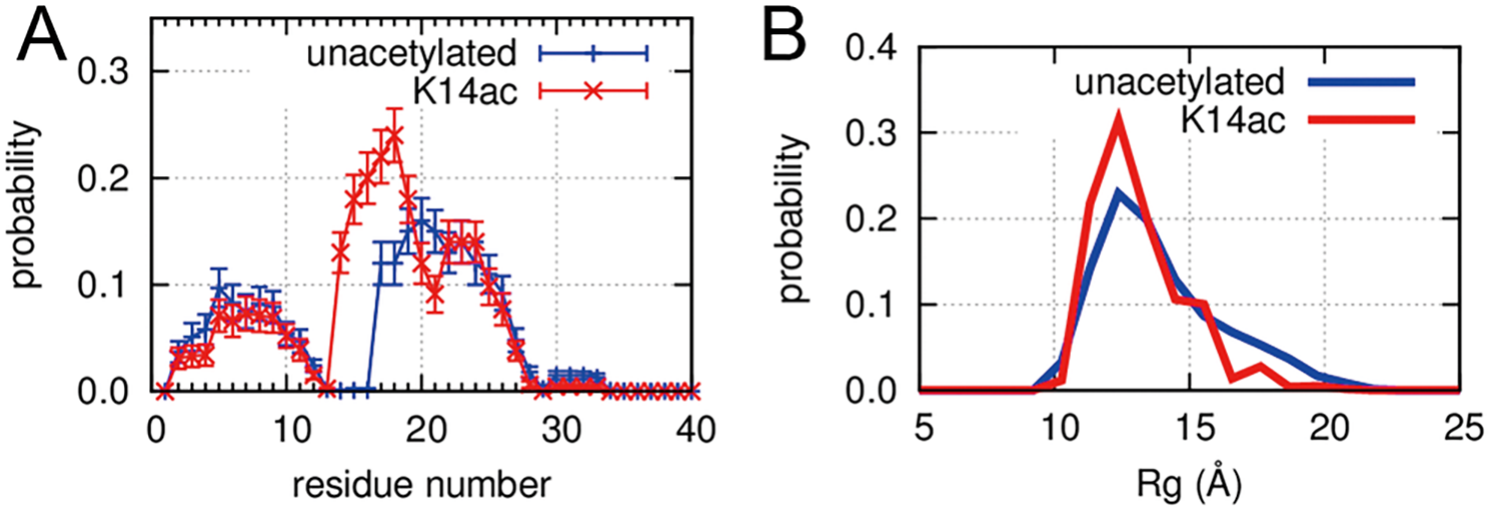
Impact of K14ac on H3 tail conformation. (A) Average α-helical content ratio with the standard error for each residue of unacetylated and K14ac H3 tails. The error bars represent the standard errors calculated from 256 independent trajectories. (B) Rg distributions for unacetylated and K14ac H3 tails.

Our simulation showed that, unlike the structured histone core domain, the H3 tail had no specific native conformation within the nucleosome, with or without K14 acetylation. Nonetheless, the H3 tail was not just a disordered region; it did have conformational preferences. To characterize the conformations of the H3 tail, we used the program DSSP[16] to analyze the secondary structure in the reweighted ensembles. Fig 2A shows the average α-helix content ratio with the standard error for each residue. Residues 2–12 and 17–28 had high helix content in the H3 tail, indicating the tail has the ability to form α-helix within the nucleosome, which is consistent with CD results from Baneres et al.[17]. It has also been reported that a tail segment isolated from H3 has the ability to form α-helix[11]. However, ours is the first MD simulation of the H3 tail within the nucleosome and confirms the H3 tail has the ability to form α-helix. Dreveny et al. [18] reported that residues 4–11 in the H3 tail form α-helix within a complex composed of the tail and histone acetyltransferase (PDB ID: 4LK9, 4LKA and 4LLB for unmodified, K9ac and K14ac H3 tail, respectively[18]). Our simulation suggests the same region to which the enzyme binds adopts an α-helical conformation, irrespective of the acetylation. In addition, the α-helix content was clearly increased with K14ac, particularly at residues 14 to 19 (Fig 2A). The increase of helical content by acetylation is consistent with previous experimental results by CD spectra [17, 19]. We depict the α-helix obtained from the conformational ensembles in S3 Fig, where two α-helices are formed in the tail.

### With K14ac the H3 tail adopts more compact conformations

We also calculated the radius of gyration (Rg) of the H3 tail. The averages and standard errors for the unacetylated and K14ac systems were 13.90 ± 0.14 and 13.14 ± 0.10Å, respectively. Although the difference was small, the K14ac H3 tail tended to be more compact than the unacetylated tail. In particular, the ratio of Rg values larger than 15 Å was lower in the tail with K14ac (Fig 2B). These results indicate that K14ac causes the H3 tail to assume a more compact conformation, which is consistent with the increase in α-helix content.

### K14ac enhances dissociation of the linker DNA from the histone core

Contact between the linker DNA and histone tail was investigated and is shown in S4 Fig, parts A and B. The unacetylated tail made contact with the DNA at the −9 to −6 and 8 to 12 bp positions (the root of the H3 tail was defined as 0 bp) more frequently than did H3 K14ac, but less frequently at the 1 to 4, and 6 bp positions (S4 Fig, part B). The K14ac tail contacts the DNA nearer to the root of the H3 tail. This is consistent with our finding that the K14ac tail adopts more compact conformations. The contour maps of the spatial distributions of the H3 tail and nucleosomal DNA (S5 Fig) were constructed as follows. Conformations from the reweighted ensembles were structurally aligned to the histone core region of a corresponding reference structure taken from the 1KX5 model without the H3 tail. We then calculated the frequencies that heavy atoms in the H3 tail, and DNA appeared in predefined 3D space grids. Consistent with the CSA result, the distributions clearly showed that the H3 tail was always located near the nucleosomal DNA in both systems, with and without K14 acetylation. In the simulations, we also observed conformations in which the linker DNA was tightly wrapped around the histone core, as was seen in the crystal structure, as well as conformations in which the linker DNA was partially dissociated from the histone core.

**Fig 3:**
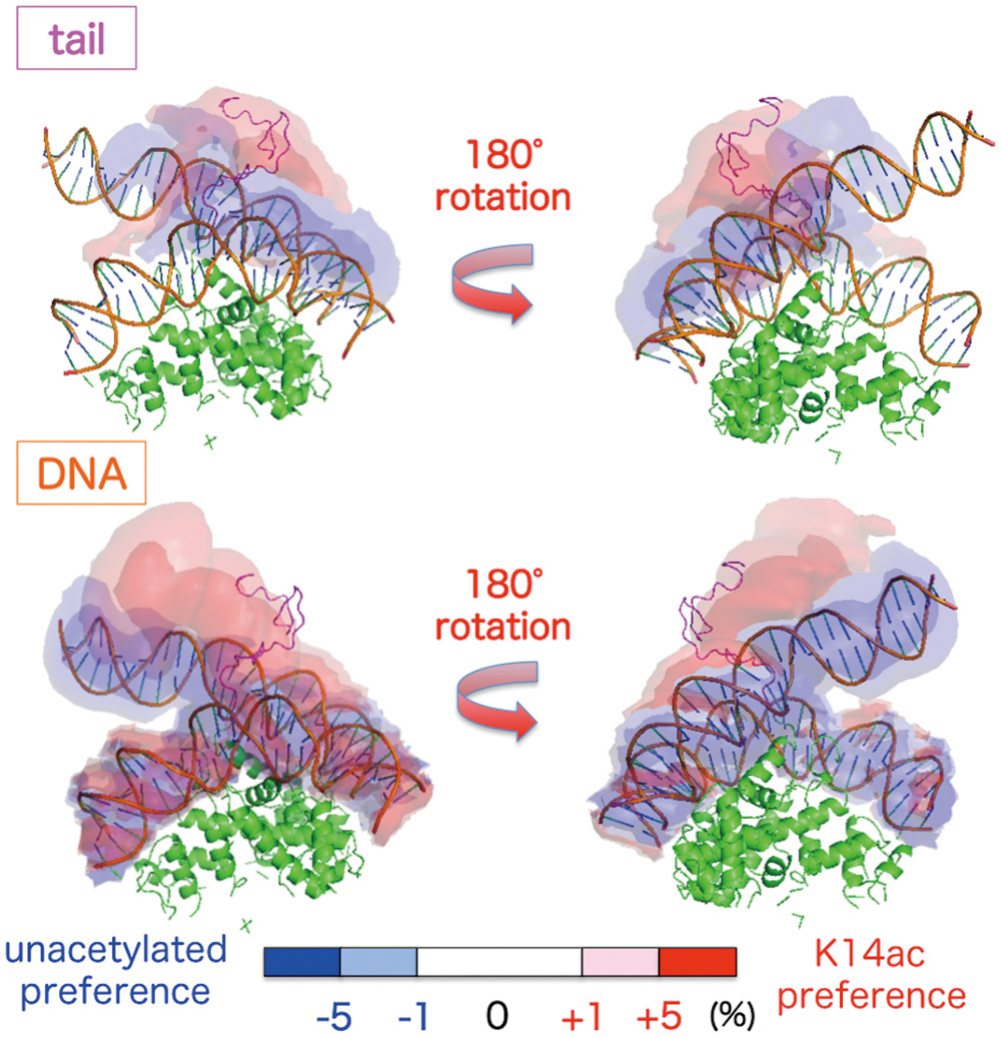
Differences in the spatial distributions between the unacetylated and K14ac systems. Differences in the H3 tails and DNA are shown in the upper and lower panels, respectively. Blue and red contour maps show spatial regions preferred in the unacetylated and K14ac system, respectively. The coloring of the molecules and the nucleosome model are the same as in Fig 1.

To determine the degree to which the H3 tail conformations differ in the presence and absence of K14 acetylation, we calculated differential maps for the spatial distributions of the DNA and H3 tail with and without acetylation of the tail (Fig 3). The difference in the distributions of the H3 tail indicates the preferences of the conformation with and without acetylation. The unacetylated tail distributed broadly along the DNA (blue region in Fig 3 top), while the K14ac tail was more likely to be distributed more compactly around the root of the tail (red region in Fig 3 top), as indicated by differences in the Rg distributions shown in Fig 2B. In addition, dissociation of the linker DNA was enhanced in the K14ac system (Fig 3 bottom, red region) as compared to the unacetylated system (Fig 3 bottom, blue region).

### Side chain of K14ac was highly exposed to the solvent

We evaluated the exposure of each residue in the H3 tail to the solvent to assess the accessibility of K14ac. Generally speaking, positively charged residues such as lysine and arginine energetically favor exposure to the solvent or contact with negatively charged molecules such as nucleosomal DNA. For acetylated lysine residues which are charge-neutralized, solvent exposure might be less energetically favorable, but they provide a binding site for transcription factors and so must be exposed to solvent.

**Fig 4:**
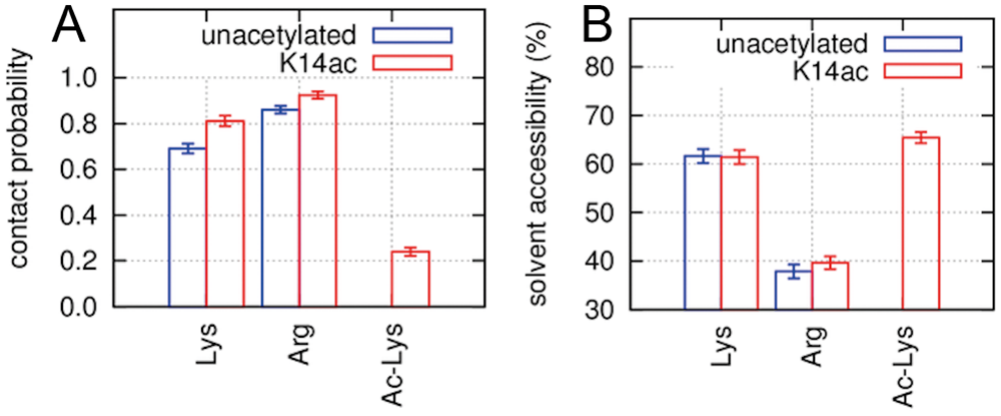
Averaged DNA contact and solvent exposure of lysine, arginine and K14ac residues within the H3 tail. (A) Contact ratios for side chain terminal atoms of lysine, arginine, and K14ac residues with DNA. (B) Exposure ratios of the atoms to solvent. The error bars represent the standard errors calculated from 256 independent trajectories.

To investigate lysine exposure with and without acetylation, we used two indices for each side chain terminal atom in lysine, arginine, and K14ac residues: the DNA contact ratio and the solvent exposure ratio. In these analyses, we considered only heavy atoms, and atomic radii used were the same ones used for the CSA analysis. The exposure ratio was defined as the relative SASA of each terminal atom to those when the side chain was fully extended. We used as terminal atoms, the NZ atom for lysine, NH1 and NH2 for arginine, and OT and CT for K14ac. Fig 4A shows that the contact ratio for K14ac was obviously lower than that for the arginine and other lysine residues, indicating that acetylation greatly reduced the contact with the DNA. Fig 4B shows the solvent exposure ratios. The exposure of K14ac was slightly higher than that of unacetylated lysine, although the difference is smaller than was observed for the contact ratio. Our simulation suggested that this is because the terminal atoms of K14ac are sometimes buried in the tail. Note that the contact ratio analysis considered contacts only with DNA atoms; other atoms in the tail were not counted. These results show that the solvent exposure of the terminal atoms of K14ac is greater than or at least equal to that of unacetylated lysine residues, which barely contacted the nucleosome DNA. Thus acetylation of K14 affects the conformations of the tail and the DNA and mediates K14 exposure to the solvent, making it available for recognition by regulatory proteins (e.g., transcriptional factors).

These analyses also revealed that there are differences between lysine and arginine residues. Fig 4 and S6 Fig show that arginine residues have consistently higher DNA contact ratios and lower solvent exposure ratios than lysine residues, irrespective of their location in the tail: arginine’s contacts with DNA are stronger than lysine’s, and lysine is easier to expose to the solvent than arginine. These characteristics may be important for the interaction between proteins and DNA. For example, it was previously shown that substituting lysine for arginine in H3 histone induces unwrapping of the nucleosome DNA[20]. Although it is generally thought that arginine and lysine have similar characteristics and lysine is sometimes substituted with arginine to see the suppressive effect of acetylation, our results suggest that such substitution might induce unexpected results due to difference in the behavior of the two amino acids.

## Discussion

Although histone tails are biologically important regions, details of their conformations in the context of nucleosomal structure are not well understood, in large part because of their conformational flexibility. This lack of structural information limits our understanding of how the tails contribute to gene regulation. In this work, we used ALSD simulations to elucidate some of the molecular details of H3 tails, with and without acetylation, and clarified the differences between their ensembles.

### Flexibility of H3 tail

NMR studies [6, 7] suggested that the flexible H3 tail extended at least to residue 35 of H3 histone. Further, Gao et al.[7] showed that the side chains of two residues, H39 and Y41, were likely immobile, and K36 to P38 were experimentally indeterminable. Our analysis of phi-psi backbone distributions agreed well with these experimental results. It suggested that residues 1 to 36 except A15, P16, A29 and P30 were flexible, while residues after K36 were immobile (S7 Fig). In addition, phi-psi plots in the simulation clearly showed an increase in α-helical content at positions 14 to 18 upon K14 acetylation.

### Mechanism of increased α-helix formation upon acetylation

Wang et al. showed that acetylation increases the α-helical content of histone tails both in the nucleosome and in solution, irrespective of their interaction with the DNA [19]. Note that in their experiment, all four core histones were acetylated to some degree. They also speculated that the increase in α-helix mediated by acetylation would limit the region of the DNA with which the tail could interact, as we inferred from our simulations. Interestingly, they showed that the increase in α-helix content in the tails at residues 14 to 18 was independent of their interaction with DNA. Consistent with those findings, our simulation demonstrates that the tail conformation causes a change in the DNA dynamics, and suggests that acetylation increases α-helical content. We inferred this increase is due to the favorable, electrostatic interaction of K14ac and K18 when adopting the α-helix. In H3 tail, Lys residues reside every four or five in sequence. These Lys residues are aligned on one side and stacked closely when the tail forms an α-helix. Acetylation of Lys removes unfavorable, electrostatic interaction between Lys residues, thereby increases the α-helical content.

### Acetylation enhances DNA dissociation

Our simulation and FRET data [21–23] both suggest that acetylation of H3 tail enhances DNA dissociation. A FRET experiment showed the acetylation of H3 tails is associated with DNA unwrapping while that of H4 tails not [21], which is consistent with our simulation result. Controversially, another FRET experiment on nucleosome core particle with a 147 bp DNA fragment showed that acetylation did not cause change in relative FRET efficiency [22]. This is also consistent with our simulation, since corresponding average distances between the bases at positions -68 and 7 in our sampled conformations with and without acetylation did not show significant difference (S8 Fig, parts A and C). Nevertheless, the distances between the linker DNA and inner DNA (at positions -83 and 0) were significantly different, suggesting that the H3 K14ac still affects the dynamics of linker DNA (S8 Fig, parts B and C).

Here, we try to speculate the dissociation mechanism based on our atomic model simulation results. In the unacetylated system, the H3 tail distributed broadly along the DNA, and it seemed to function as a scaffold between the linker DNA and the nucleosome core region, preventing the unwrapping. In the acetylated system, the H3 tail became more compact in the region around the root of the tail, and the tail formed a conformational cluster with positive charges on the surface of the nucleosome. This cluster may elevate the linker DNA, inducing the unwrapping from the histone core. Our simulation results provide a molecular evidence that the neutralization of the K14 charge by acetylation affects the DNA configuration by changing the tail conformation and the point of contact with the DNA, though the H3 tail itself does not dissociate from the DNA.

### Future direction

Our simulation, as well as other NMR and FRET studies, suggests the structural impact of acetylation is subtle at the single nucleosome level, but it is sufficient to change the specificity of the PTM recognition site. The next step in simulation is to study chromatin aggregation and dissociation. What happens when hyper-acetylation or accumulation of acetylation occurs within chromatin? A coarse-grained simulation is one way to study such phenomena.

MD simulation is a promising approach to understanding the details of conformational states, but it remains beyond the computational power currently available to apply all-atom MD to study the entire chromatin structure. Instead, MD simulations based on coarse-grained models simplify the atomic representation to reduce the computational cost [24–28]. When carrying out such simulations, it is key to determine appropriate force field parameters for histone tails. There are no perfect coarse-grained parameters that can apply to all of biological systems. To stabilize the native conformation in a coarse-grained MD, parameters like Go-potential specialized for each system are often introduced into the simulations. Our simulation results provide information that could be utilized to determine such parameters using, for example, the inverse Monte Carlo method [29].

In our simulation, the tail did not behave as a Gaussian chain and always had contacts with the nucleosomal DNA. This indicates that histone tails do not behave like a random chain, but instead have conformational preferences. In addition, there are constraints on where the tail can be located. Development of a model that takes these features into account is necessary. Schlick’s group has already moved in that direction with their coarse-grained model [24–26]. We also suggest development of a force field that reflects the difference between lysine and arginine residues, as our simulation showed arginine interacted with DNA to a greater degree than Lys, and Lys was more exposed to the solvent. In most coarse-grained MD employing a one-residue one-particle model, lysine and arginine are represented as similar residues with the same positive charge and different vdW radii. The expression of this difference is important in an epigenetic point of view.

### Conclusion

We performed conformational sampling simulations of histone H3 tails, with and without K14 acetylation, within the nucleosome structure to characterize the conformational states and study the impact of acetylation. The obtained conformational ensemble showed that 1) H3 tails with or without acetylation are located nearby the nucleosomal DNA and maintain contact with the DNA, and 2) parts of the H3 tail formed α-helix irrespective of K14 acetylation, but the tendency to form helix is stronger in the acetylated system. Acetylation slightly weakened the interactions between the tail and the DNA due to the charge neutralization and made the tail conformation more compact. We suggest the compaction induces unwrapping of the linker DNA so that transcriptional factors can access the DNA. In addition, we demonstrated differences in the characteristics of arginine, lysine and acetylated lysine, suggesting the necessity to develop a new force field for coarse-grained simulation that reflects these differences.

## Methods

### Simulation model

To obtain a conformational ensemble of histone H3 tails within a nucleosomal structure, we performed a simulation with a system that included both the H3 tail and the linker DNA. No nucleosome structures that included the atomic coordinates of both regions have been deposited in the PDB. We therefore constructed the system using two nucleosome models, 1KX5[15] and 1ZBB[30]. 1KX5 is a mono-nucleosome model with modeled histone tails. 1ZBB is a tetra-nucleosome model with linker DNA. Using those models, we constructed the hybrid nucleosome model shown in Fig 1. Histones from 1KX5 and DNA from 1ZBB were combined by superimposing their common histone core regions. In this way, we extended the DNA by 10 bp at the end, as compared to 1KX5. Then to reduce computational cost, only atoms within a sphere of 54 Å radius at the root of the H3 tail (a nitrogen atom in the 40-th residue) were considered in the simulations. The N- and C-termini of the trimmed amino acid residues were capped with acetyl and N-methyl groups, respectively. The system was then immersed in a sphere of explicit water: the center was the same as the sphere described above, the radius was 60 Å and the water molecules were equilibrated at 300 K and 1 g/cc in advance. Water molecules overlapping histones and DNA were removed. We exchanged some water molecules with Na^+^ and Cl^-^ ions to neutralize the net charge and bring the ion concentration close to physiological (0.153 M). Ultimately, the system consisted of 90306 atoms (3554 atoms for DNA, 5886 atoms for histone, 34 chloride ions, 111 sodium ions, and 80721 atoms for water) for the unacetylated system (Fig 1). A second system for K14ac was also constructed in the same manner (total 90306 atoms, 3554 atoms for DNA, 5890 atoms for histone, 33 chloride ions, 111 sodium ions, and 80718 atoms for water).

### Force fields and the MD simulation procedure

We used force field parameters taken from an AMBER-based hybrid force field (ω = 0.75) [31], AMBER bsc0[32], TIP3P [33], and ion08[34] for the proteins, DNA, water molecules and ions, respectively. We used point charge parameters published by Papageorgiou on the Web (http://pc164.materials.uoi.gr/dpapageo/amberparams.php) for acetylated lysine. To maintain the trimmed nucleosome conformation, weak harmonic potentials as position constraints referring to the 1KX5 model were applied to heavy atoms in the vicinity of the water sphere boundary. Note that the position constraints were not applied to the linker DNA. To maintain hydrogen bonds for base pair formation, we also applied weak harmonic potentials as distance constraints. To avoid evaporation of water molecules from the water sphere boundary shown in Fig 1, a harmonic potential (force constant = 100 kcal/mol/Å^2^) was applied to the water oxygen atoms only when they were outside the boundary. We used the MD simulation program PRESTO ver. 3[35] extended by the authors. A time step of 2 fs was used. The SHAKE algorithm[36] was used to constrain the geometry of atom groups X-H (X is a heavy atom). The cell-multipole expansion method[37] was used to compute long-range electrostatic interactions, and the constant-temperature method[38] was applied to control the simulation temperature. We performed ALSD simulations[12] to realize an efficient conformational sampling of histone H3 tail. To speed up the sampling, we used trivial trajectory parallelization (TTP)[14], which searches a conformational ensemble with *N* (= 256, in this work) multiple, independent simulations starting from different initial conformations. We carried out iterative and productive runs for 36 ns × 256 runs = 9.216 μs and 30 ns × 256 runs = 7.680 μs, respectively (see the next section for the details of the iterative and productive runs).

### Adaptive lambda square dynamics (ALSD) simulation

ALSD[12] is a simulation technique to enhance conformational sampling in a predefined partial system, while conformations in the rest of the system were allowed to thermally fluctuate. For the ALSD procedure, we divided a system into two regions: a tail region (N-terminal 40 residues of a histone H3) and the rest (the nucleosome without the H3 tail, ions and water molecules). The total potential energy of the system, *E*, was decomposed into three terms,

$$E=E_{\text{tail}}+E_{\text{tail}-\text{rest}}+E_{\text{rest}},$$ (1)

where *E*_tail_, *E*_tail-rest_ and *E*_rest_ denote potential energy terms for intra-tail, inter tail-rest, and intra-rest regions, respectively. ALSD simulation is a canonical MD simulation with ALSD Hamiltonian,

$$H_{\text{ALSD}}=\lambda^{2}E_{\text{tail}}+\lambda E_{\text{tail}-\text{rest}}+E_{\text{rest}}+K+m_{\lambda }{\dot{\lambda }}^{2}/2+RT\text{ln}P\left(\lambda ,T\right),$$ (2)

where λ is an extra dynamic variable with the fictitious mass *m*_λ_, *K* is the kinetic energy of the system, λ is the velocity on the λ axis, *R* is the gas constant, *T* is the constant simulation temperature (in this work, *T* = 300 K), and *P*(λ, *T*) is a canonical probability distribution at λ. During the ALSD simulation, the variable λ moves on the λ axis, obeying *H*_ALSD_ and scales only the potential energy terms related to the tails. When 0 < λ < 1, interactions only for the tail are reduced, and conformational changes in the tail are enhanced. The last energy term is an umbrella potential to regulate the sampled λ range (0.6 < λ < 1.03 in this work). ALSD can realize a random walk on the λ axis if a priori unknown function *P*(λ, *T*) in the umbrella potential term is accurately estimated. The random walk facilitates overcoming energy barriers such as strong attractive electrostatic interactions between the tail and the DNA. In practical ALSD simulations, iterative runs of simulations are carried out to accurately estimate *P*(λ, *T*) before productive runs to obtain a conformational ensemble. In this work, we did 19 iterative runs each for the unacetylated and K14ac systems. A canonical ensemble at λ = 1 can be reconstructed using a reweighting scheme[12]. Unless otherwise stated, we used canonical ensembles reweighted at λ = 1 for conformational analyses.

## Supporting Information

S1 Fig Distribution of contact surface area (CSA) between the H3 K14ac tail and DNA is plotted as a function of scaling factor λ for ALSD. (TIF)

S2 Fig H3 conformation at CSA = 0 obtained from the ensemble in the K14ac system. The H3 histone tail, DNA and histone core regions are shown in magenta, orange and green, respectively.
(TIF)

S3 Fig A conformation forming α-helix obtained from the ensemble in the K14ac system. The H3 histone tail, DNA and histone core regions are shown in magenta, orange and green, respectively.
(TIF)

S4 Fig Probability the H3 tail conformation will touch the DNA in the ensemble at T = 300 K. (A) Probabilities for K14ac and unacetylated systems. (B) Probability difference between K14ac and unacetylated systems (B). The abscissa represents the relative position in base pairs from the base pair closest to the root of tail. Positive numbers indicate the direction is toward the dyad, while negative numbers indicate the direction is toward the DNA end. The errors were calculated using 256 independent trajectories.
(TIF)

S5 Fig Spatial distributions of the H3 tails (magenta) and DNA (orange) in 3D space. The distributions in the unacetylated and K14ac systems are shown in the upper and lower panels, respectively. The coloring of the molecules is the same as in S2 Fig, and the hybrid nucleosome model described in the Methods section is superimposed as a reference.
(TIF)

S6 Fig DNA contact and solvent exposure of lysine, K14ac and arginine residues within the H3 tail. (A) Contact ratios of the side chain terminal atoms of lysine, arginine and K14ac with DNA. (B) Exposure ratios of the atoms to the solvent. The error bars represent the standard errors calculated from 256 independent trajectories. The background colors of green, blue and red denote lysine, arginine and K14ac, respectively.
(TIF)

S7 Fig Ramachandran plots for the backbone of residues 2 to 40. (PDF)

S8 Fig Distance distributions between two bases for the re-weighted ensembles. (A) Distance between two bases corresponding to the FRET experiments [22]. The average distance was 28.01 (std. error ± 0.08) Å for the unacetylated system and 28.00 (std. error ± 0.06) Å for the K14ac system, respectively. (B) Distance between two bases in the linker DNA and inner DNA. The average distances were 48.89 (std. error ± 0.29) Å and 50.39 (std. error ± 0.31) Å. (C) The location of the bases used in distance measurement is shown by two cyan spheres for (A) and two magenta ones for (B), respectively. Note that the distances plotted are shorter than R0 in FRET experiments because the distance in FRET experiment is the distance where FRET efficiency is 50% between two dye molecules. Also, the distance depends on the dye molecules’ conformation.
(TIF)
