# Supplementary figures and images for: H3 Histone Tail Conformation within the Nucleosome and the Impact of K14 Acetylation Studied Using Enhanced Sampling Simulation
Source: PLoS Comput Biol. 2016 Mar 11;12(3):e1004788. doi: 10.1371/journal.pcbi.1004788 (PMC4788430; doi:10.1371/journal.pcbi.1004788)

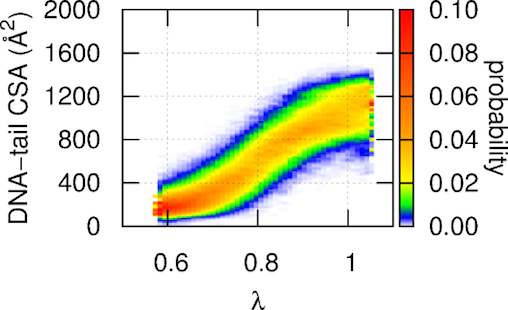

Supplement: S1 Fig — (TIF) [file pcbi.1004788.s001.tif]

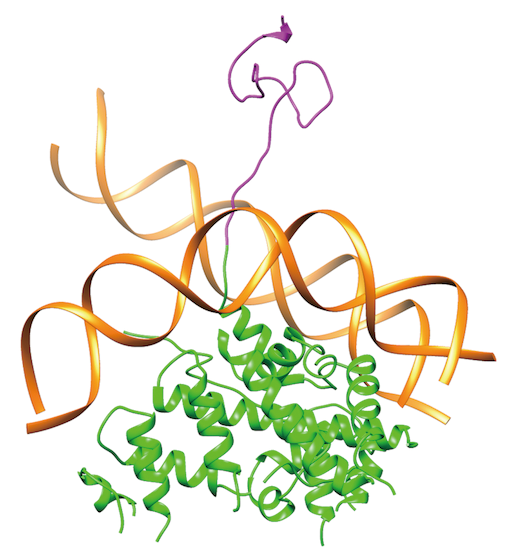

Supplement: S2 Fig — The H3 histone tail, DNA and histone core regions are shown in magenta, orange and green, respectively. (TIF) [file pcbi.1004788.s002.tif]

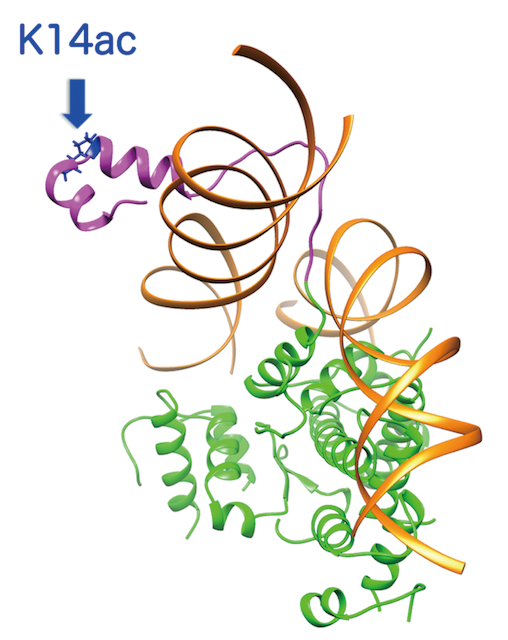

Supplement: S3 Fig — The H3 histone tail, DNA and histone core regions are shown in magenta, orange and green, respectively. (TIF) [file pcbi.1004788.s003.tif]

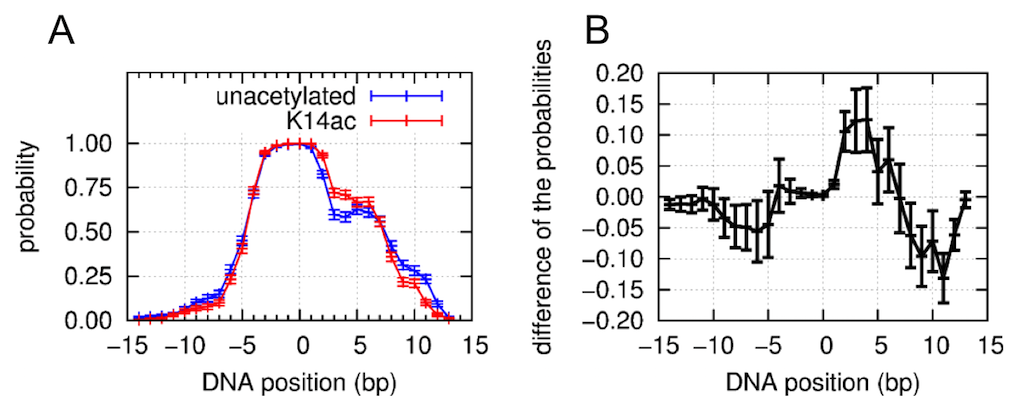

Supplement: S4 Fig — (A) Probabilities for K14ac and unacetylated systems. (B) Probability difference between K14ac and unacetylated systems (B). The abscissa represents the relative position in base pairs from the base pair closest to the root of tail. Positive numbers indicate the direction is toward the dyad, while negative numbers indicate the direction is toward the DNA end. The errors were calculated using 256 independent trajectories. (TIF) [file pcbi.1004788.s004.tif]

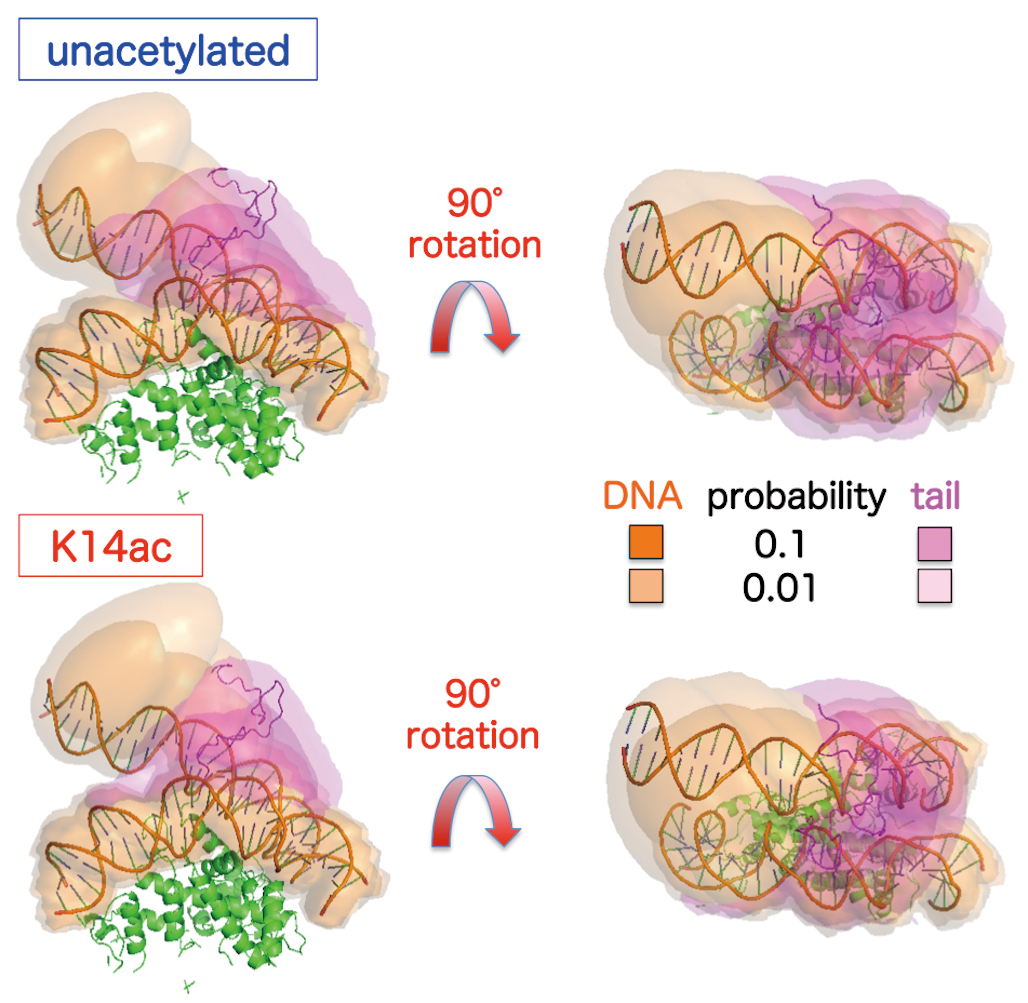

Supplement: S5 Fig — The distributions in the unacetylated and K14ac systems are shown in the upper and lower panels, respectively. The coloring of the molecules is the same as in S2 Fig, and the hybrid nucleosome model described in the Methods section is superimposed as a reference. (TIF) [file pcbi.1004788.s005.tif]

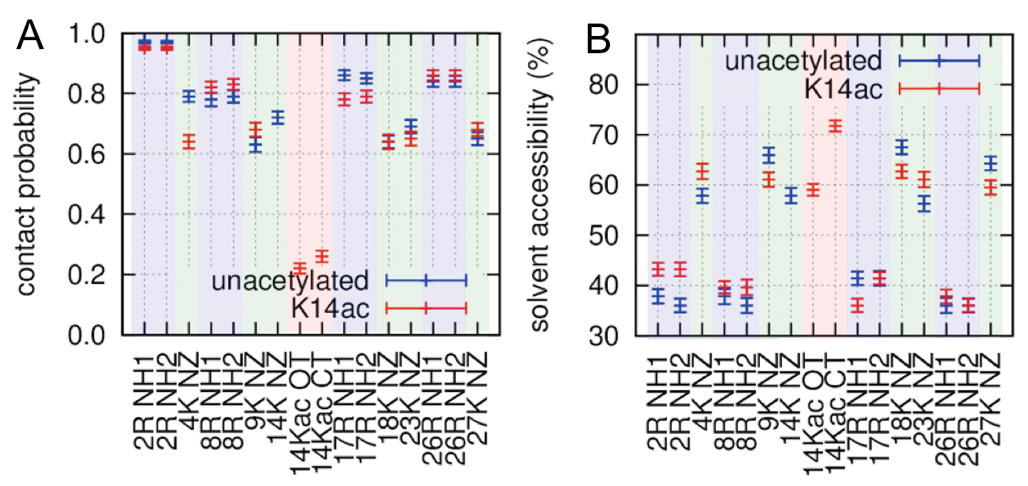

Supplement: S6 Fig — (A) Contact ratios of the side chain terminal atoms of lysine, arginine and K14ac with DNA. (B) Exposure ratios of the atoms to the solvent. The error bars represent the standard errors calculated from 256 independent trajectories. The background colors of green, blue and red denote lysine, arginine and K14ac, respectively. (TIF) [file pcbi.1004788.s006.tif]

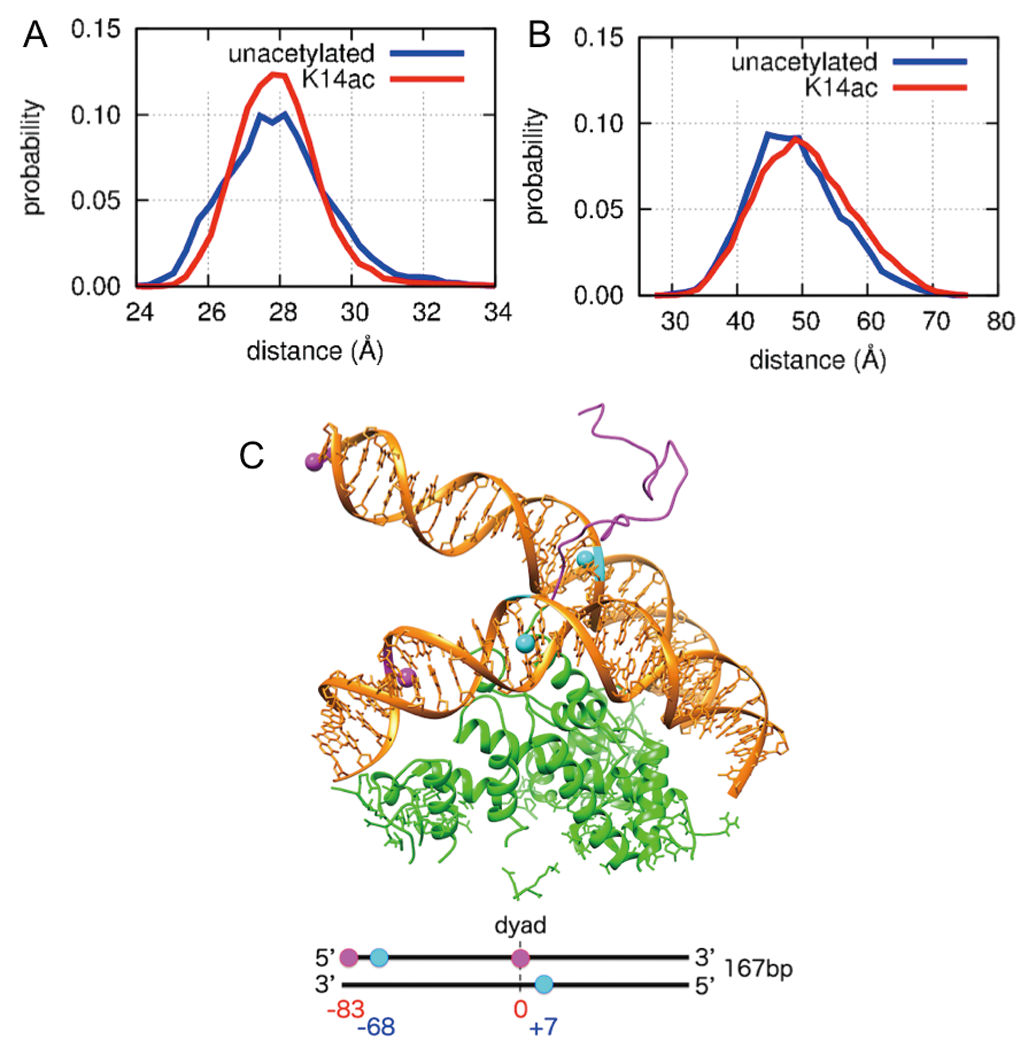

Supplement: S8 Fig — (A) Distance between two bases corresponding to the FRET experiments [22]. The average distance was 28.01 (std. error ± 0.08) Å for the unacetylated system and 28.00 (std. error ± 0.06) Å for the K14ac system, respectively. (B) Distance between two bases in the linker DNA and inner DNA. The average distances were 48.89 (std. error ± 0.29) Å and 50.39 (std. error ± 0.31) Å. (C) The location of the bases used in distance measurement is shown by two cyan spheres for (A) and two magenta ones for (B), respectively. Note that the distances plotted are shorter than R0 in FRET experiments because the distance in FRET experiment is the distance where FRET efficiency is 50% between two dye molecules. Also, the distance depends on the dye molecules’ conformation. (TIF) [file pcbi.1004788.s008.tif]
