# Supplementary material for: H3 Histone Tail Conformation within the Nucleosome and the Impact of K14 Acetylation Studied Using Enhanced Sampling Simulation
Source: PLoS Comput Biol. 2016 Mar 11;12(3):e1004788. doi: 10.1371/journal.pcbi.1004788 (PMC4788430; doi:10.1371/journal.pcbi.1004788)
Supplement: S7 Fig — (PDF) [file pcbi.1004788.s007.pdf]

2 ARG+

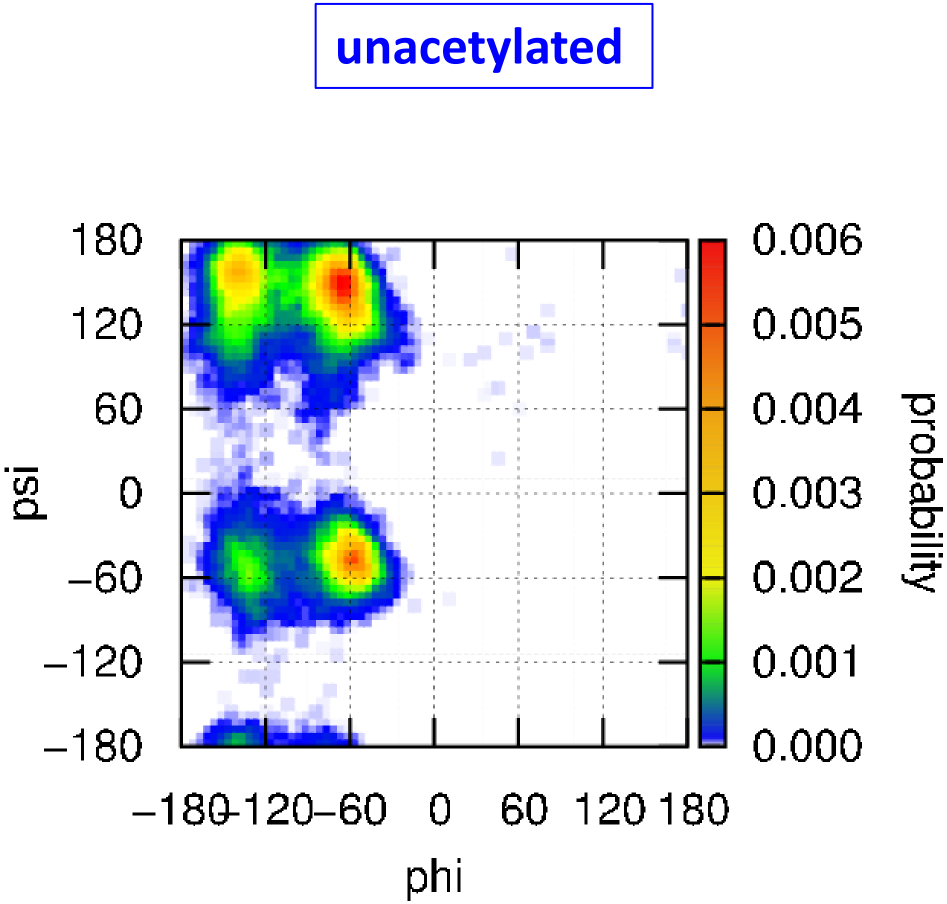
unacetylated
180
120
60
0
−60
−120
−180
psi
−180 −120 −60 0 60 120 180
phi
0.006
0.005
0.004
0.003
0.002
0.001
0.000
probability

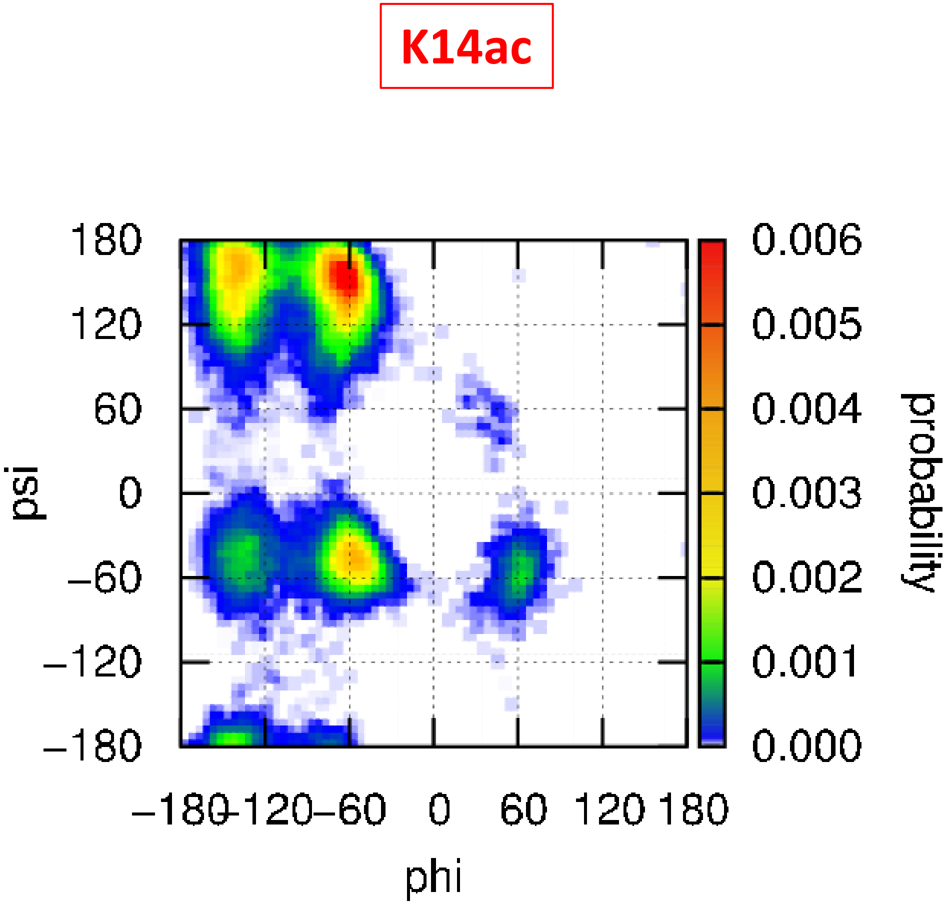
K14ac
180
120
60
0
−60
−120
−180
psi
−180 −120 −60 0 60 120 180
phi
0.006
0.005
0.004
0.003
0.002
0.001
0.000
probability

3 THR

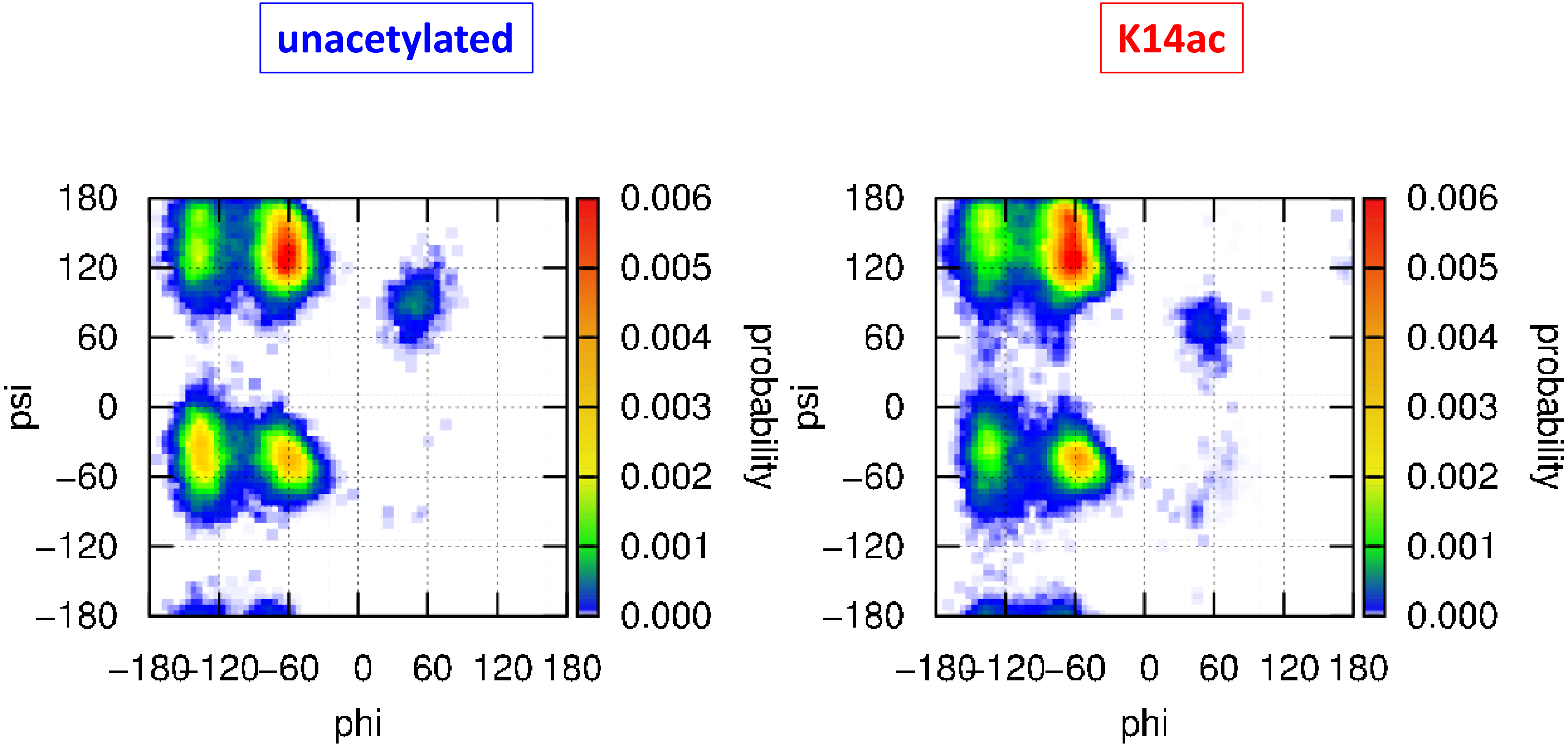

unacetylated
K14ac
180
120
60
0
−60
−120
−180
psi
−180 −120 −60 0 60 120 180
phi
0.006
0.005
0.004
0.003
0.002
0.001
0.000
probability

4 LYS+

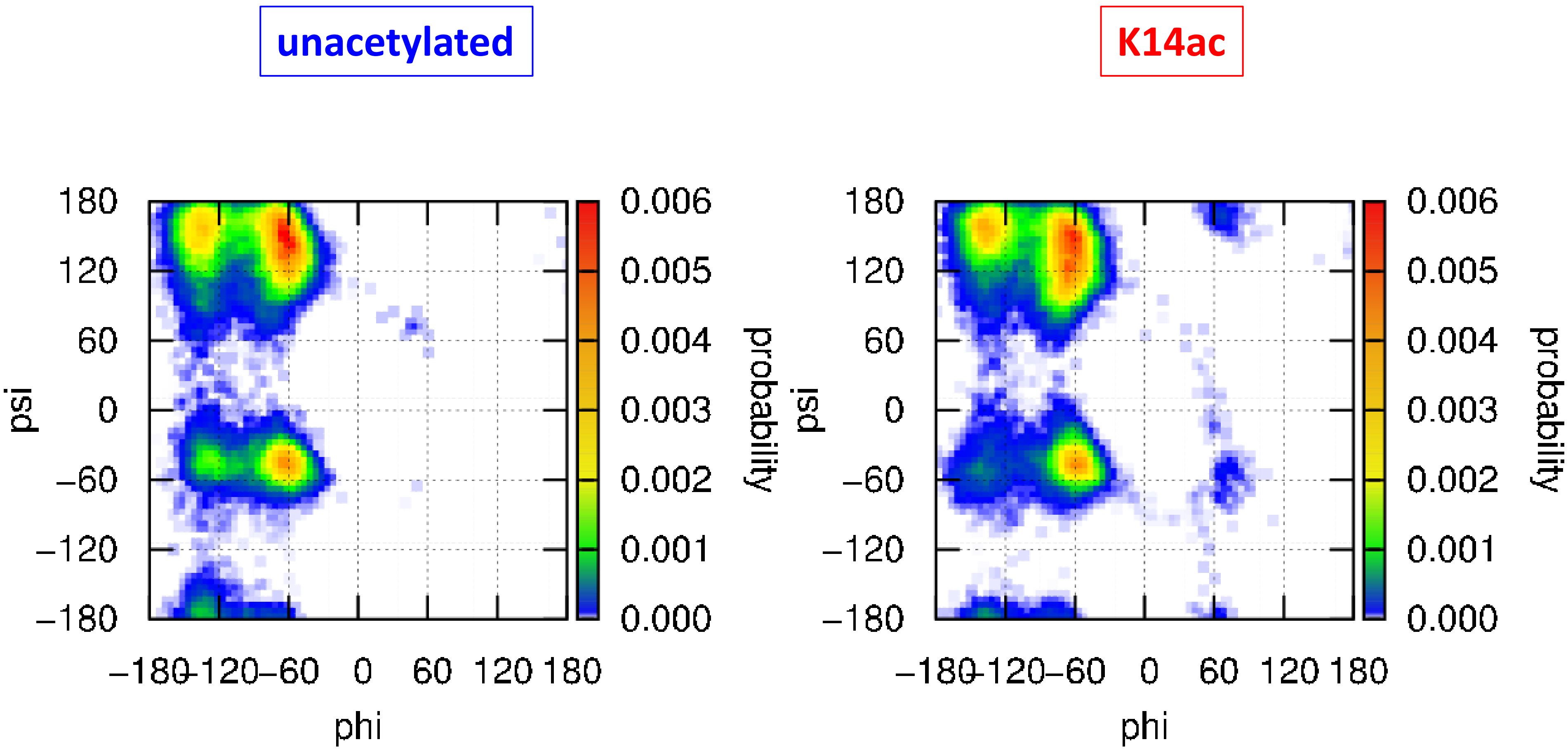

unacetylated
K14ac
180
120
60
0
−60
−120
−180
psi
−180 −120 −60 0 60 120 180
phi
0.006
0.005
0.004
0.003
0.002
0.001
0.000
probability

5 GLN

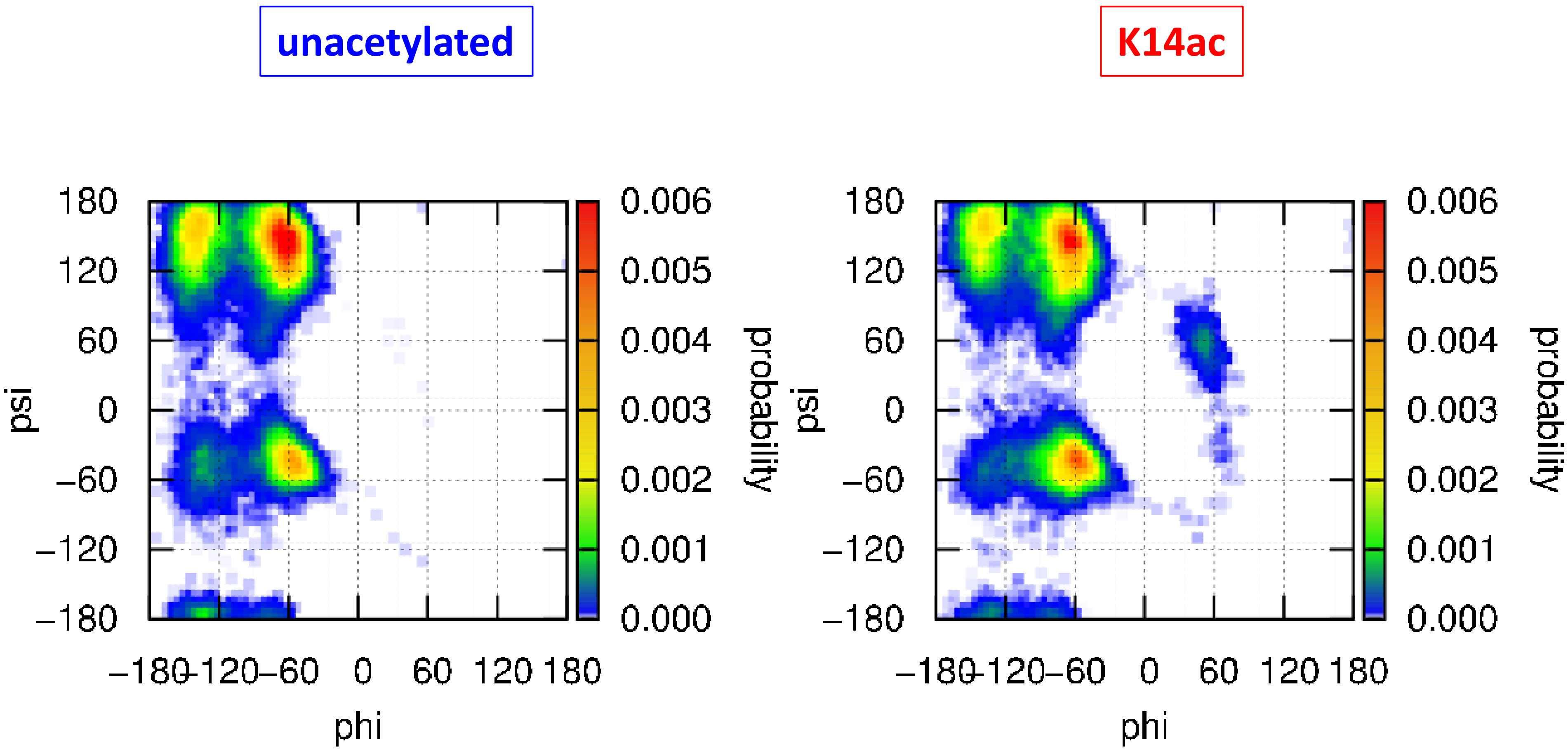
unacetylated
K14ac
180
120
60
0
−60
−120
−180
psi
−180
−120
−60
0
60
120
180
phi
0.006
0.005
0.004
0.003
0.002
0.001
0.000
probability

6 THR

unacetylated

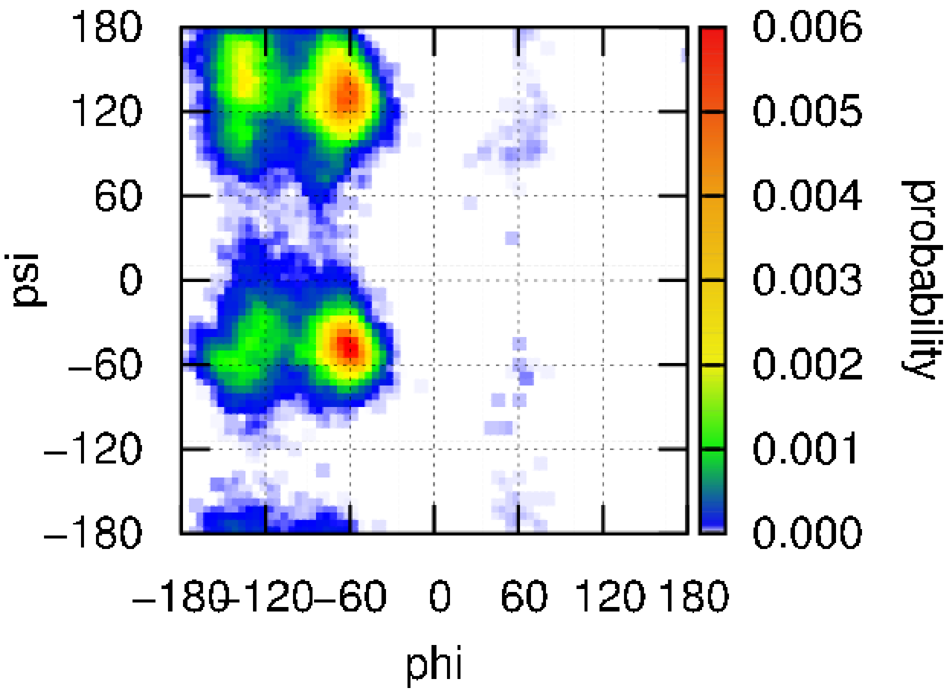

K14ac

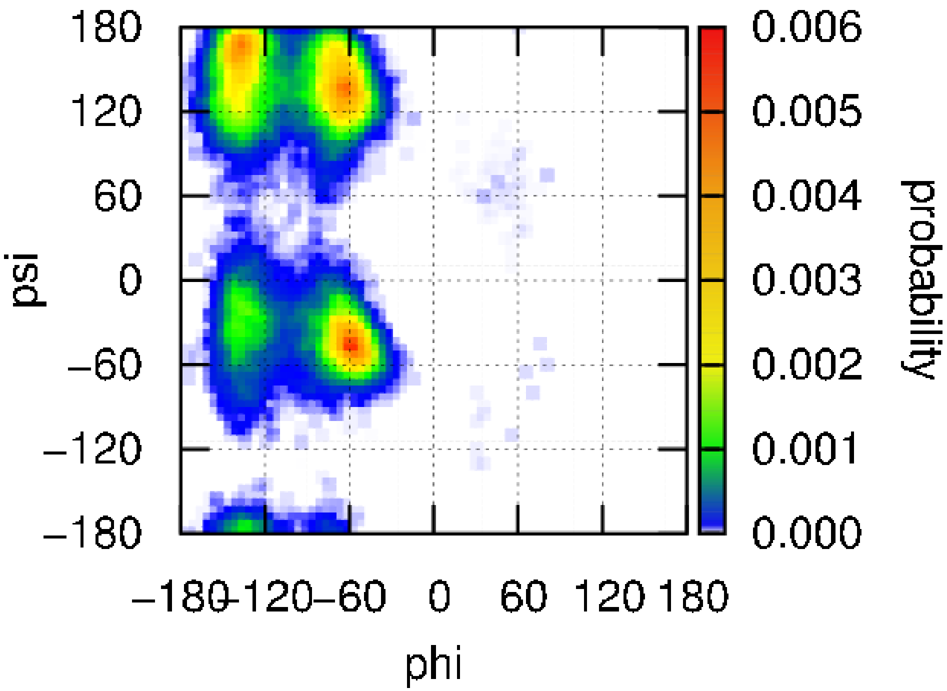

7 ALA

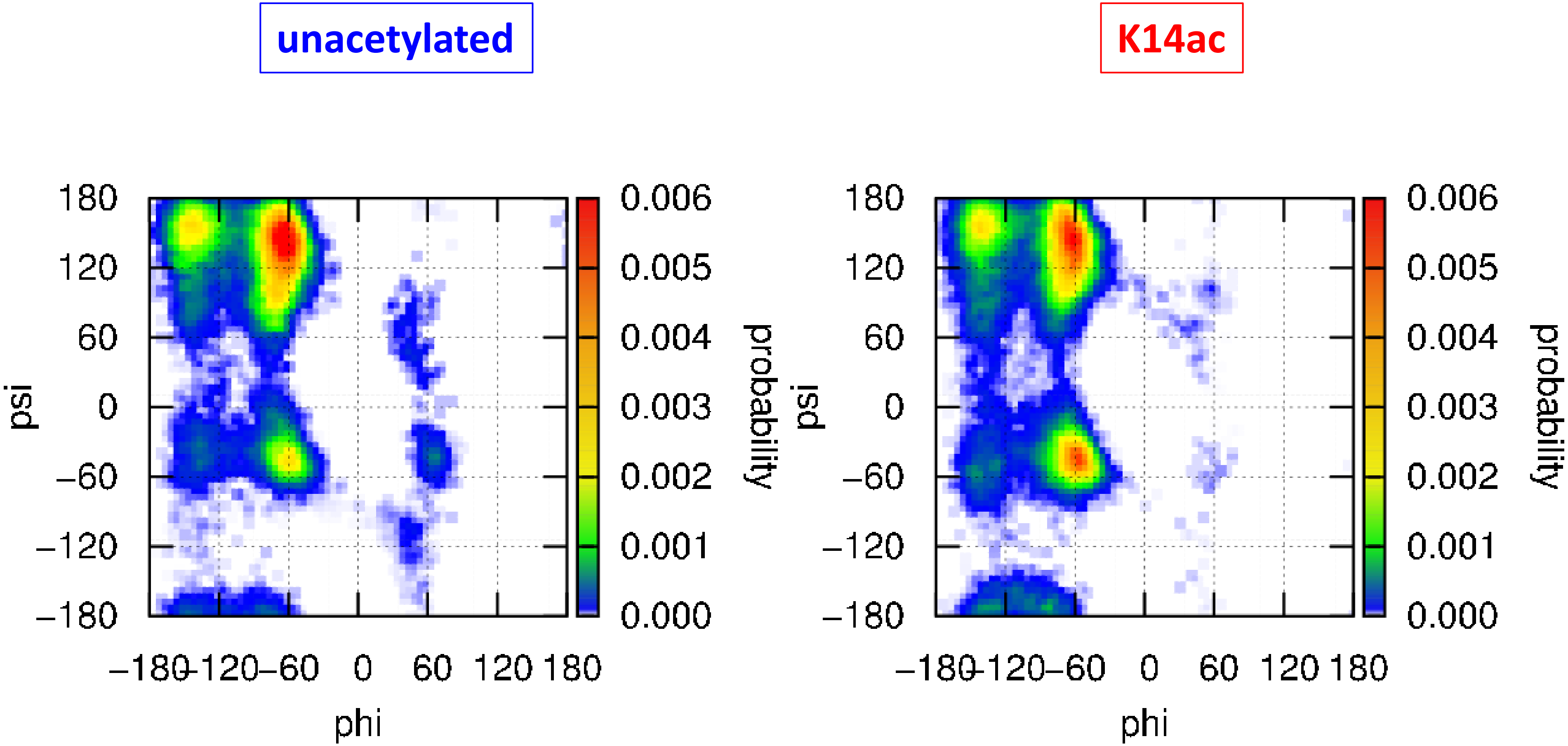

unacetylated
K14ac
180
120
60
0
−60
−120
−180
psi
−180−120−60 0 60 120 180
phi
0.006
0.005
0.004
0.003
0.002
0.001
0.000
probability

8 ARG+

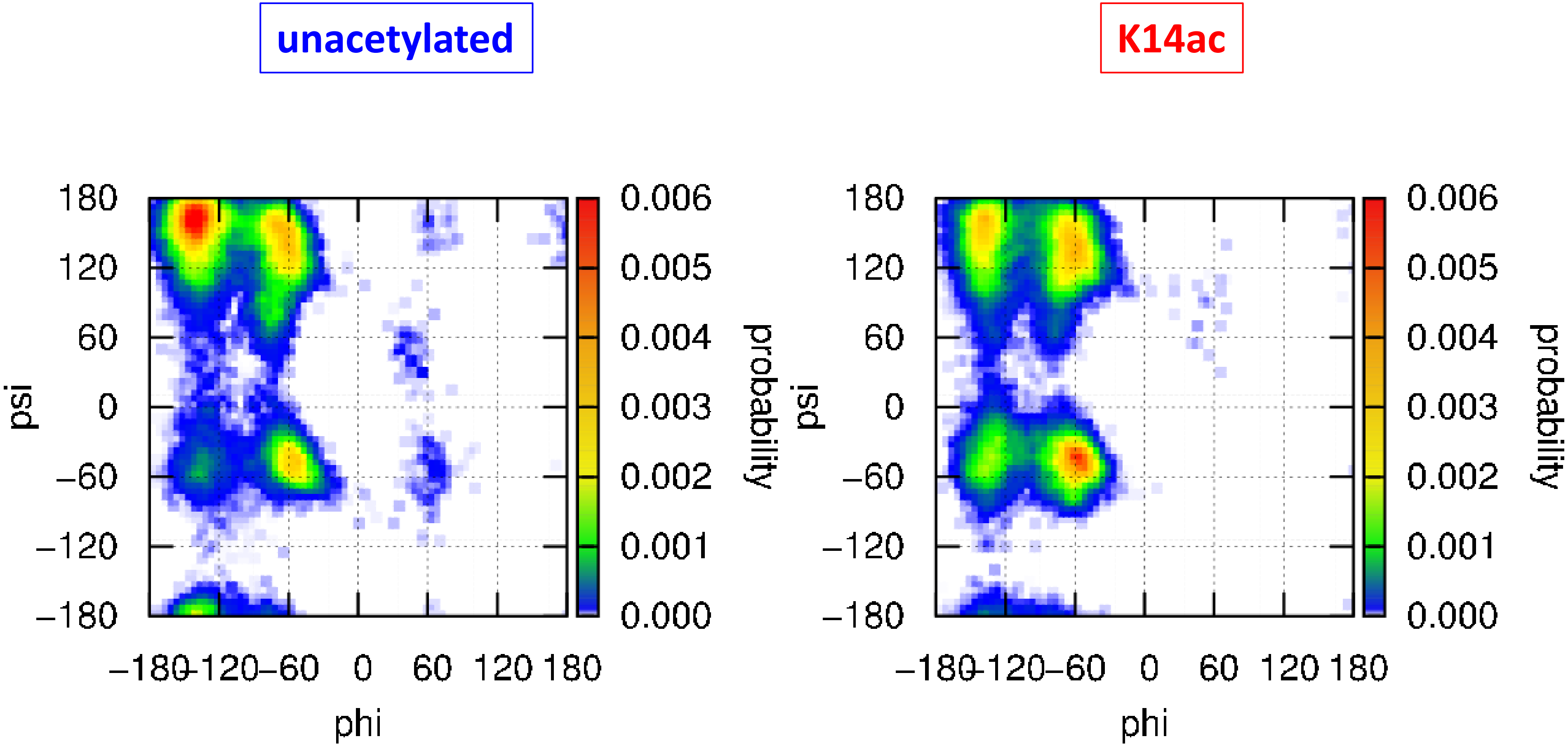
unacetylated
K14ac
180
120
60
0
−60
−120
−180
psi
−180 −120 −60 0 60 120 180
phi
0.006
0.005
0.004
0.003
0.002
0.001
0.000
probability

9 LYS+

unacetylated

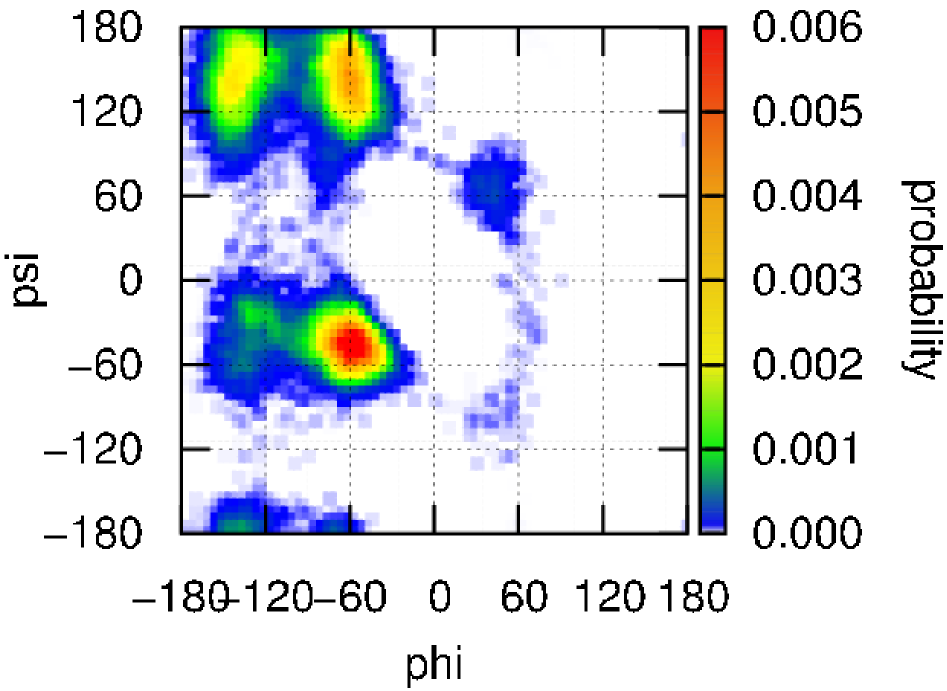

K14ac

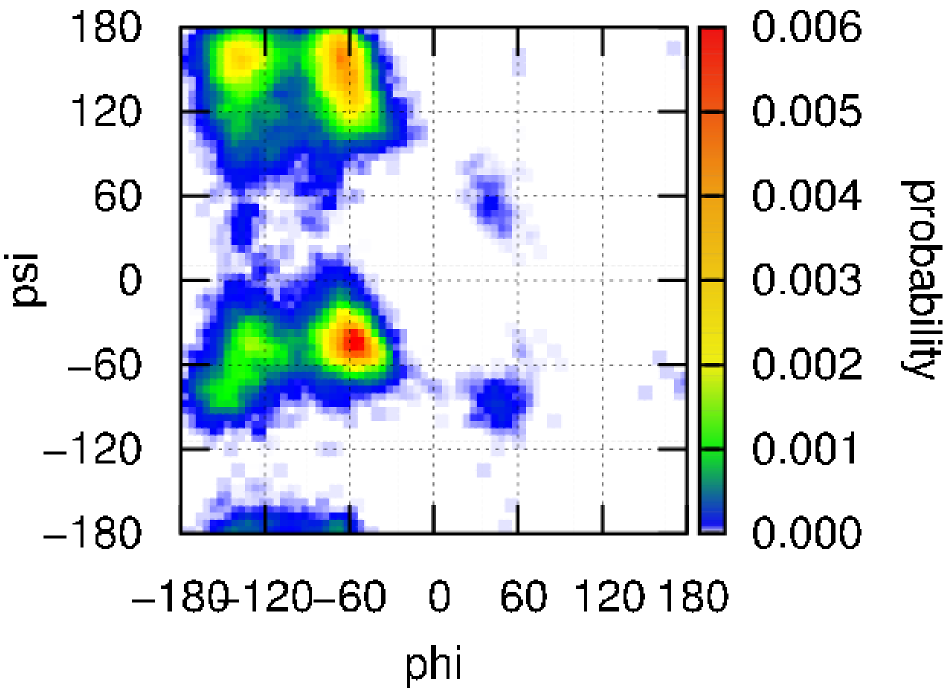

10 SER

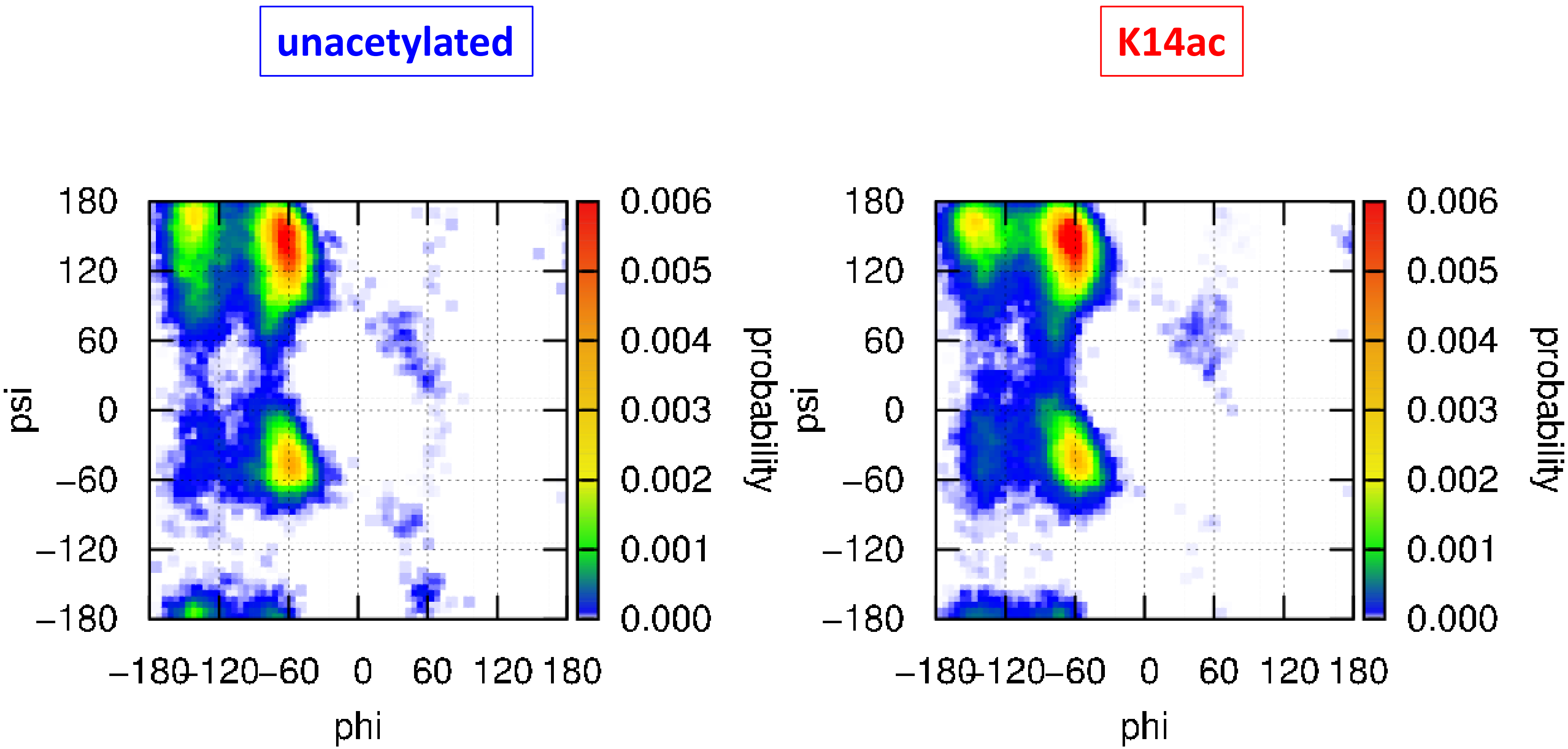
unacetylated
K14ac
180
120
60
0
−60
−120
−180
psi
−180−120−60 0 60 120 180
phi
0.006
0.005
0.004
0.003
0.002
0.001
0.000
probability

11 THR

unacetylated

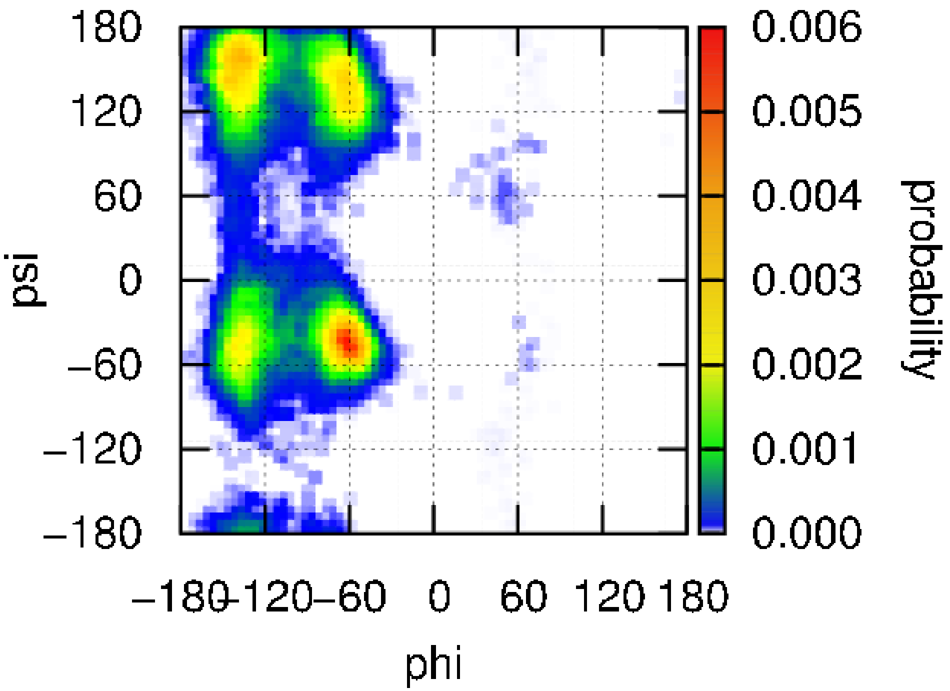

K14ac

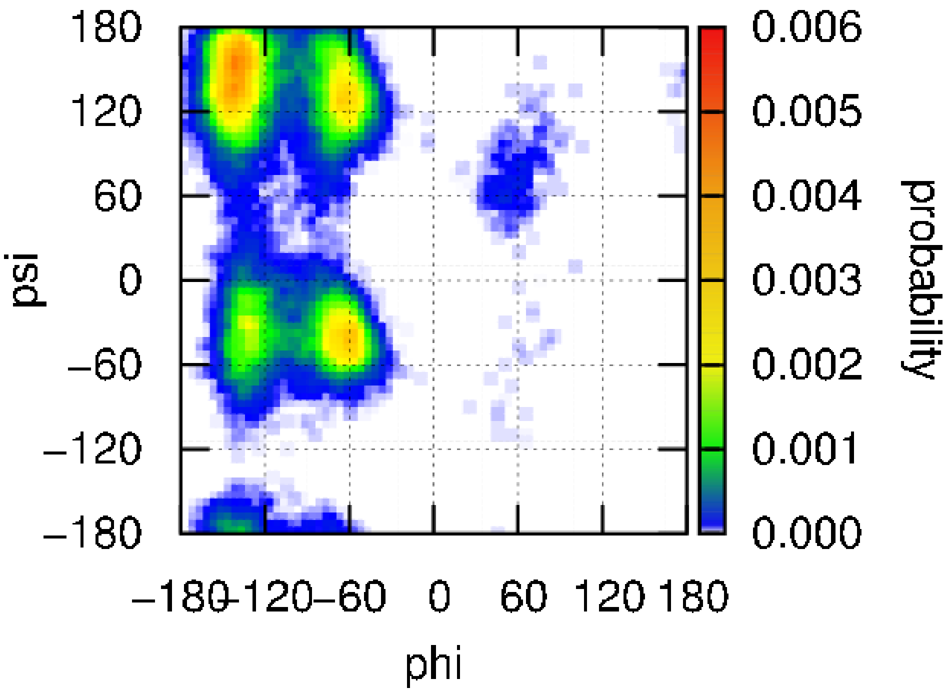

12 GLY

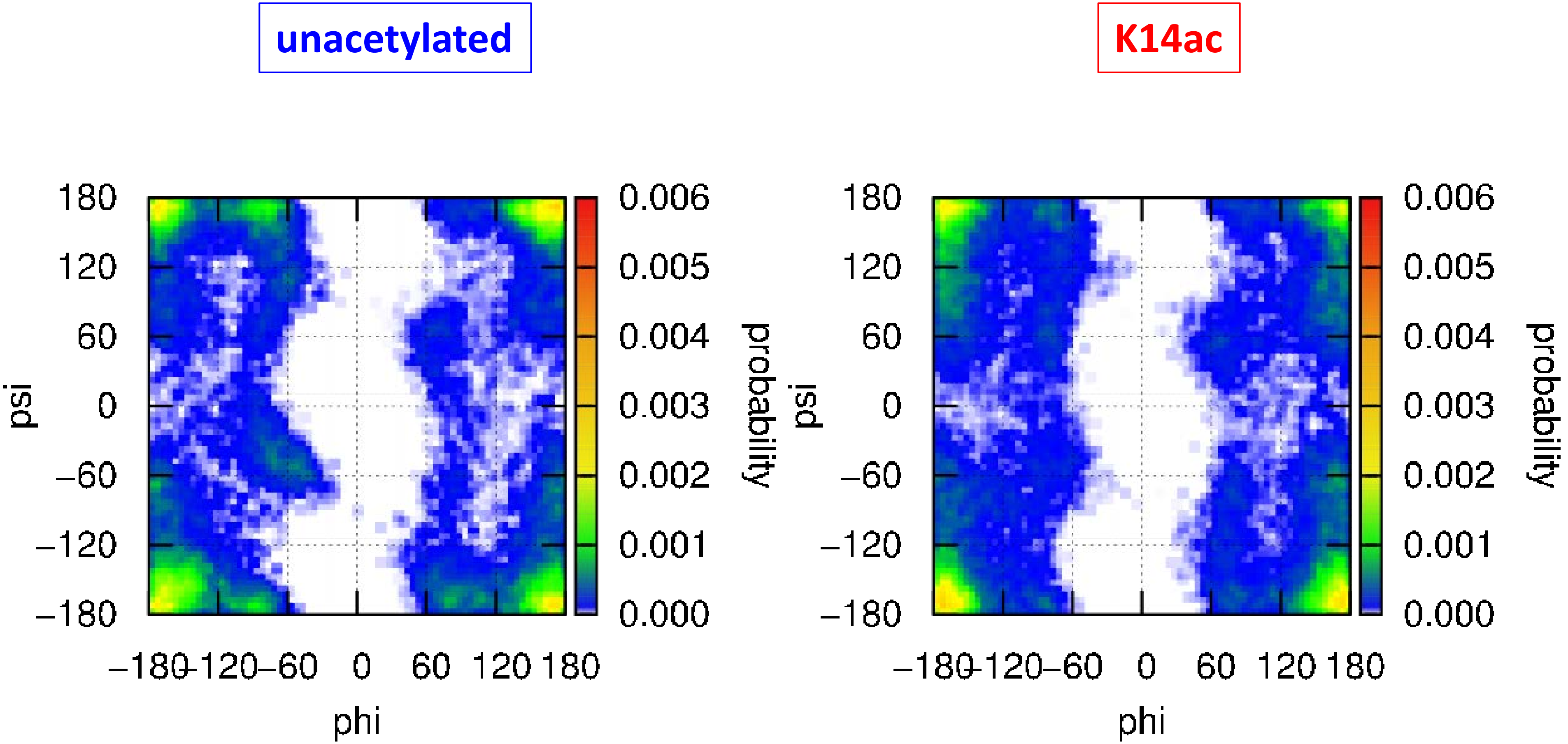

unacetylated
K14ac
180
120
60
0
−60
−120
−180
psi
phi
−180 −120 −60 0 60 120 180
0.006
0.005
0.004
0.003
0.002
0.001
0.000
probability

13 GLY

unacetylated

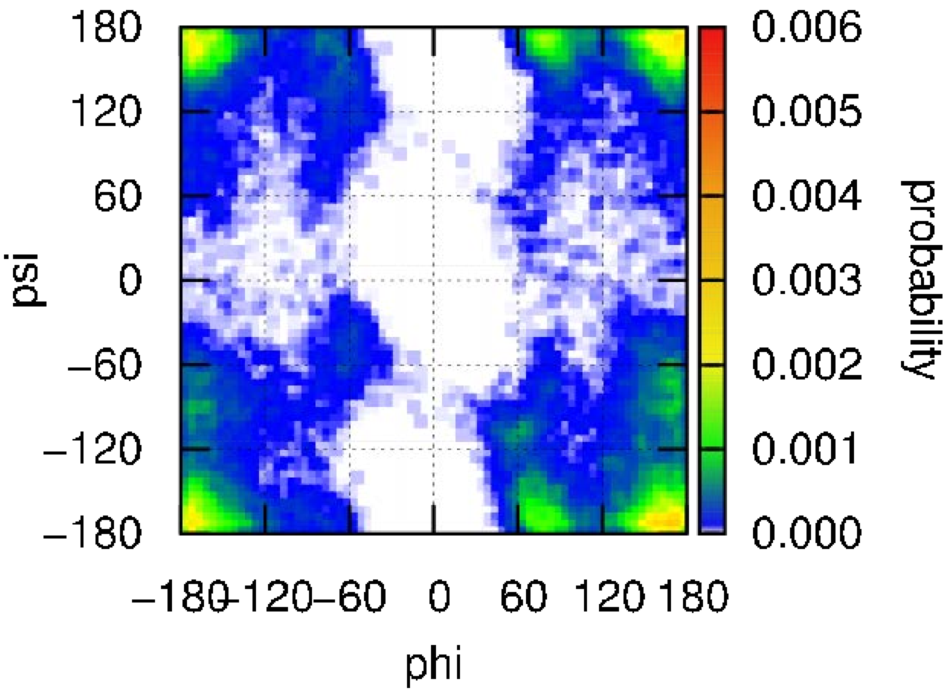

K14ac

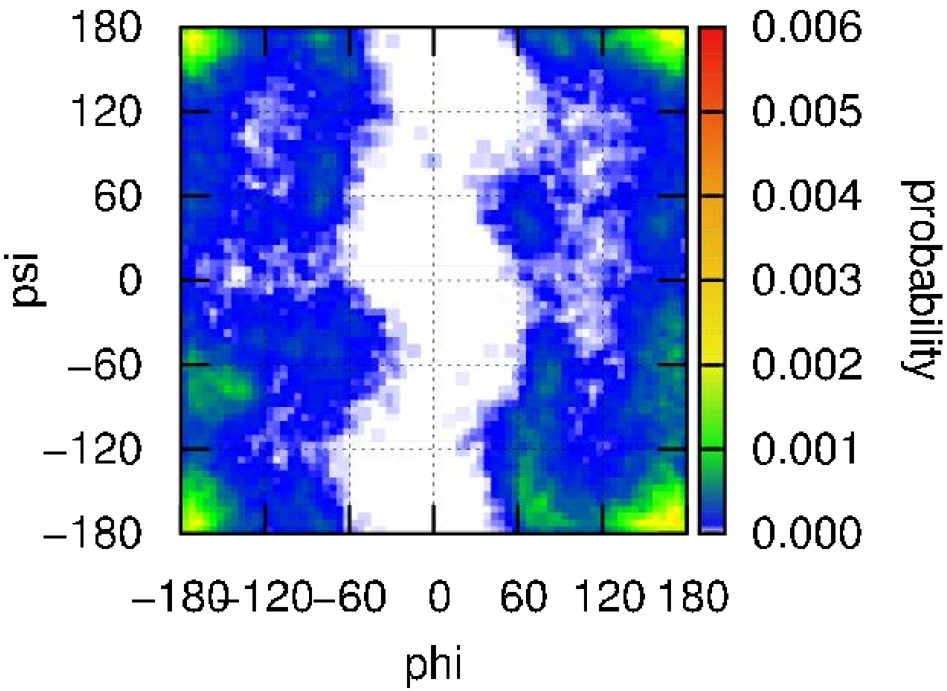

14 LYS+ or ACK

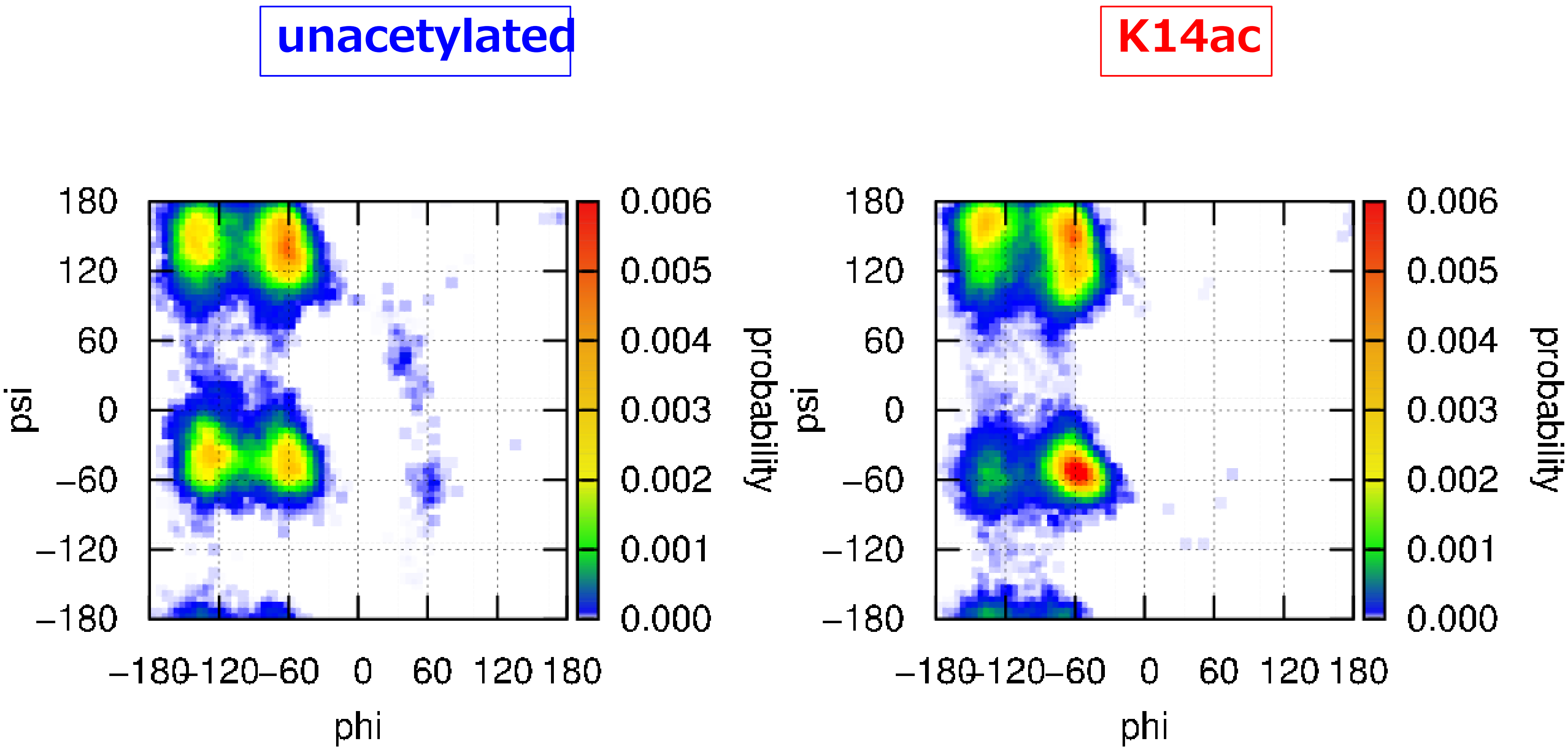

unacetylated
K14ac
180
120
60
0
−60
−120
−180
psi
−180 −120 −60 0 60 120 180
phi
0.006
0.005
0.004
0.003
0.002
0.001
0.000
probability

15 ALA

unacetylated

K14ac

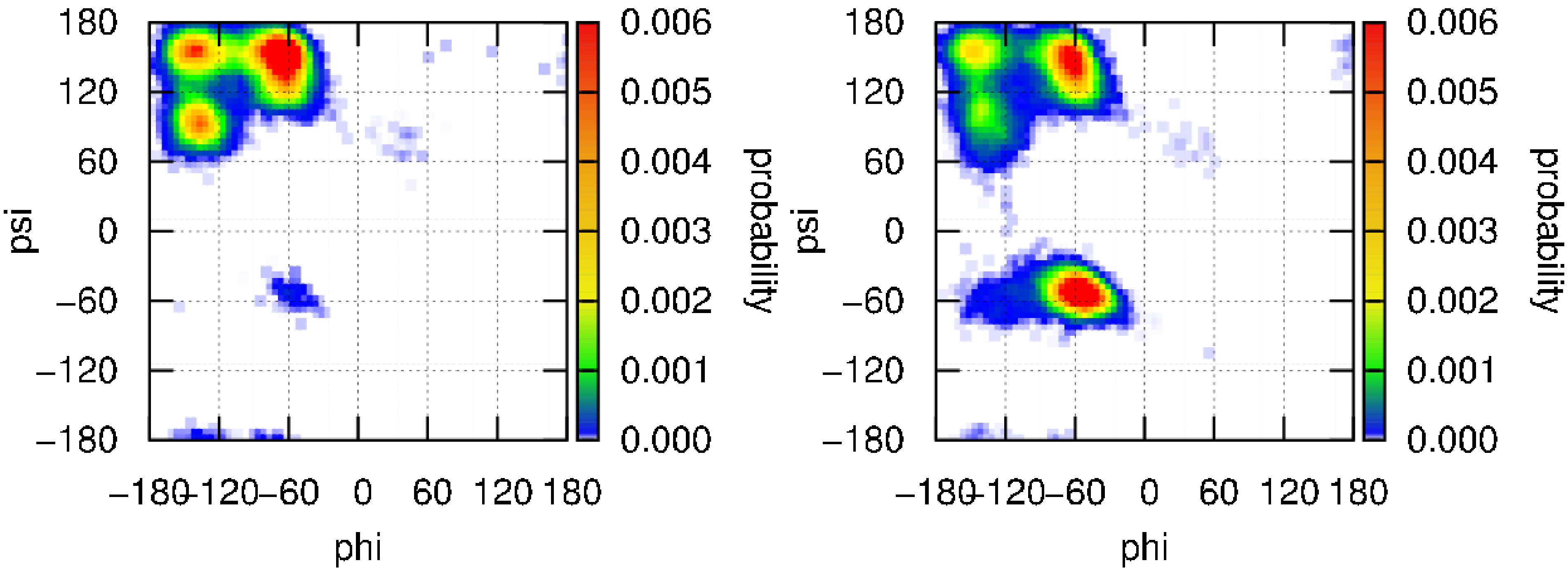

16 PRO

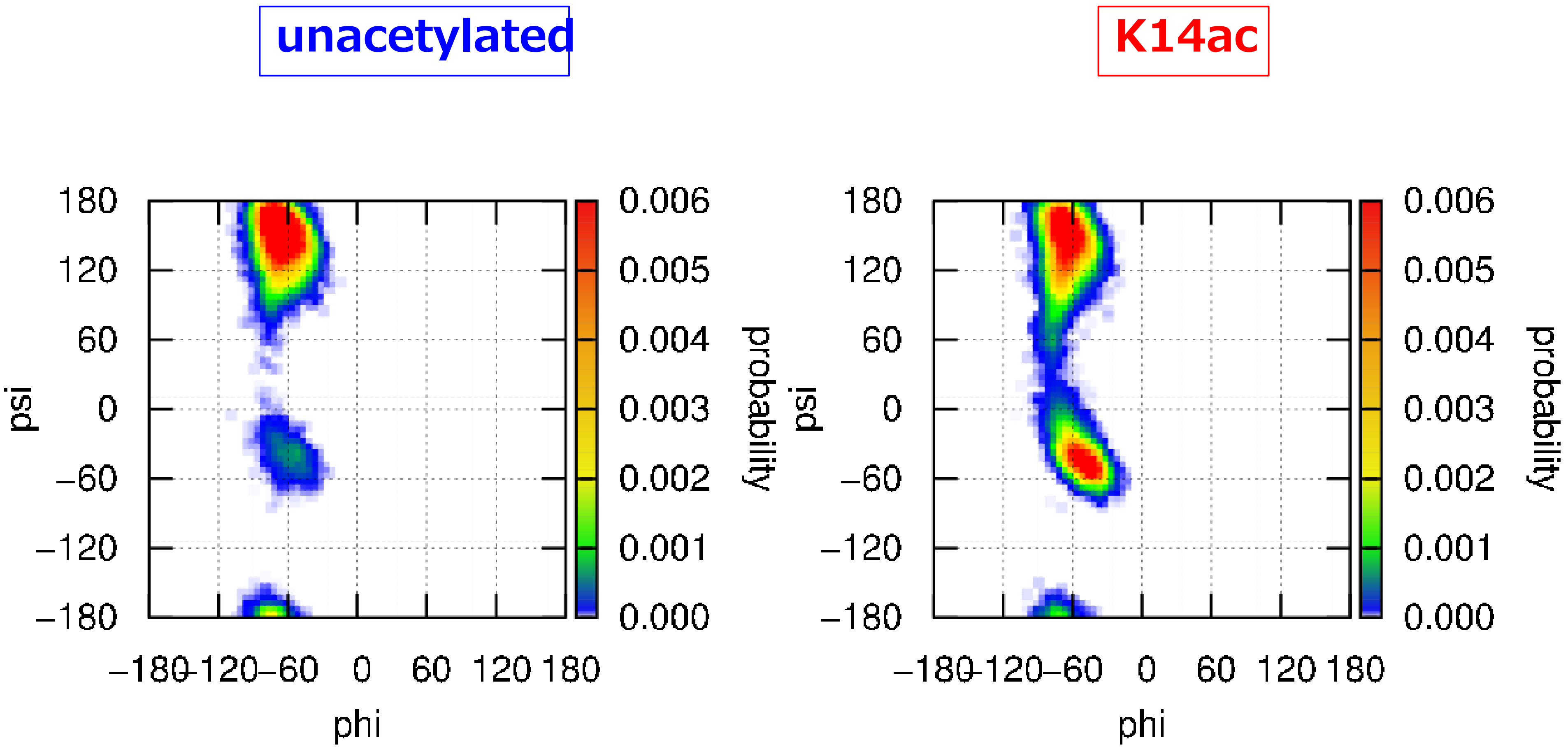

unacetylated
K14ac
180
120
60
0
−60
−120
−180
psi
−180
−120
−60
0
60
120
180
phi
0.006
0.005
0.004
0.003
0.002
0.001
0.000
probability

17 ARG+

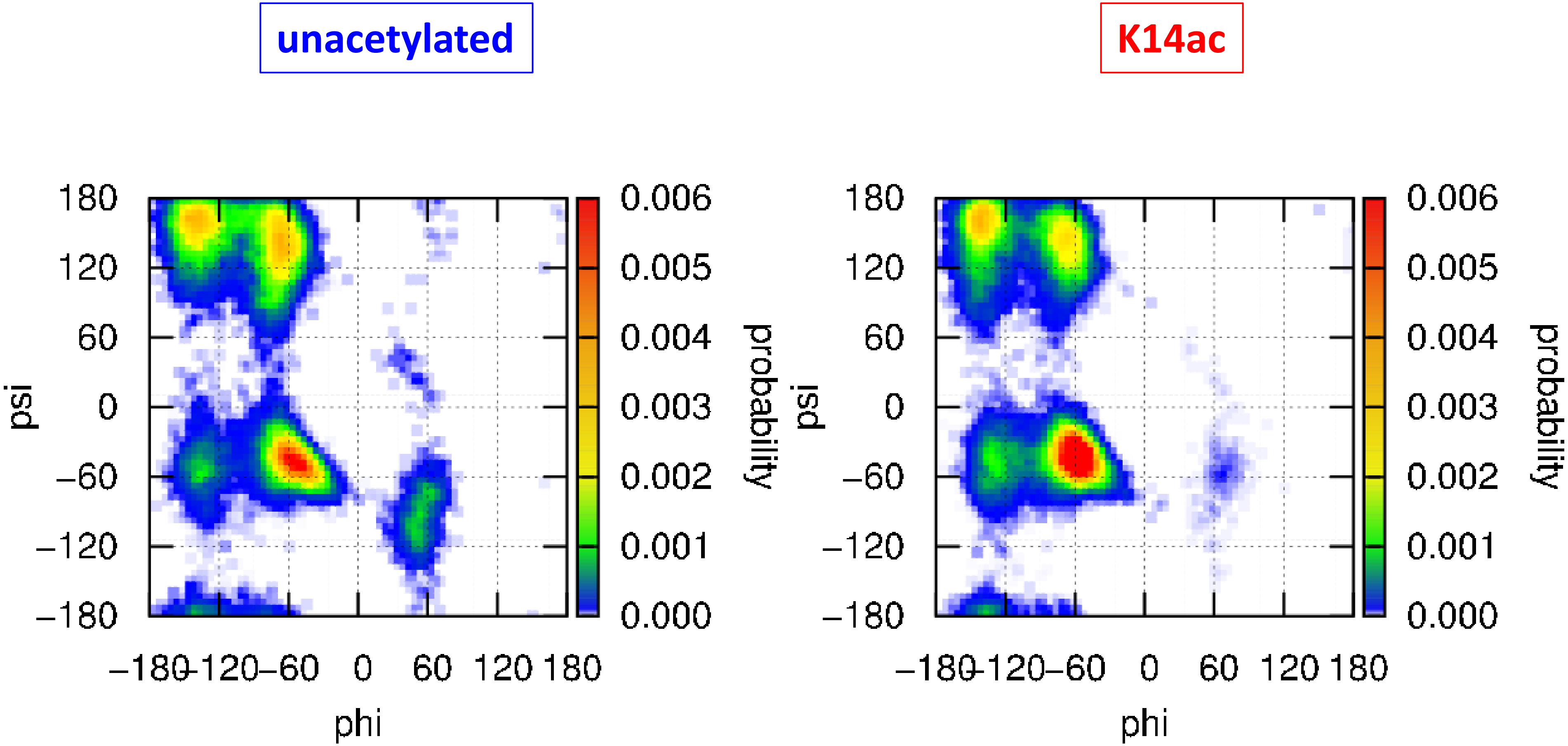
unacetylated
K14ac
180
120
60
0
−60
−120
−180
psi
−180
−120
−60
0
60
120
180
phi
0.006
0.005
0.004
0.003
0.002
0.001
0.000
probability

18 LYS+

unacetylated

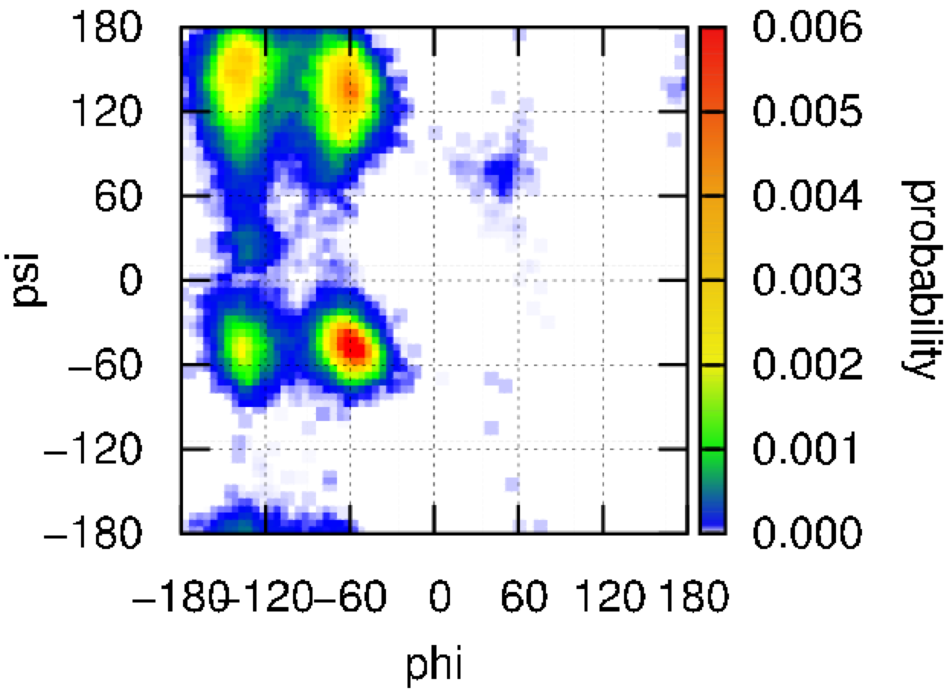

K14ac

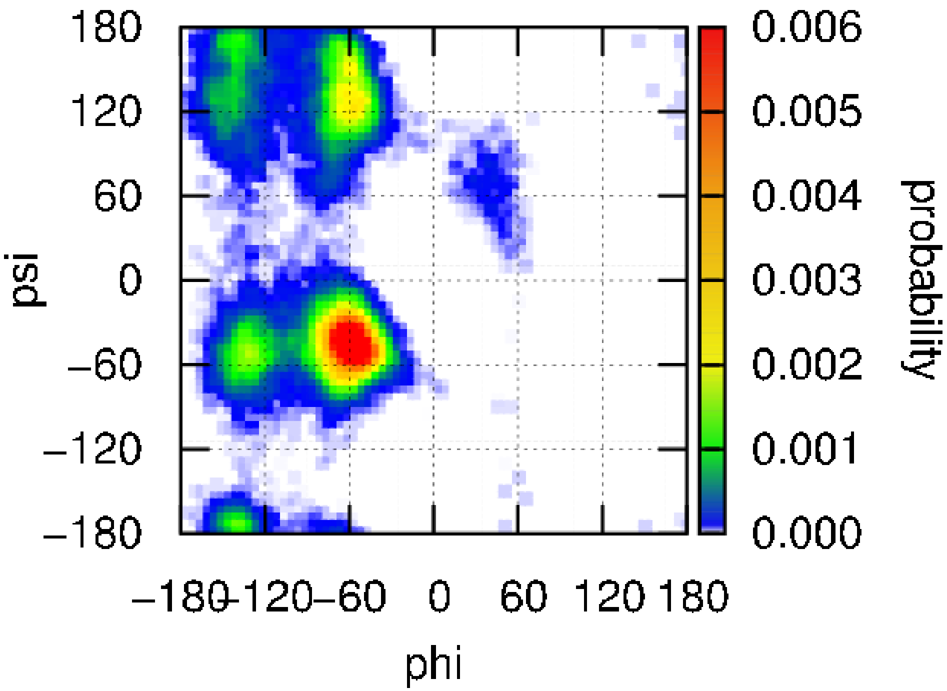

19 GLN

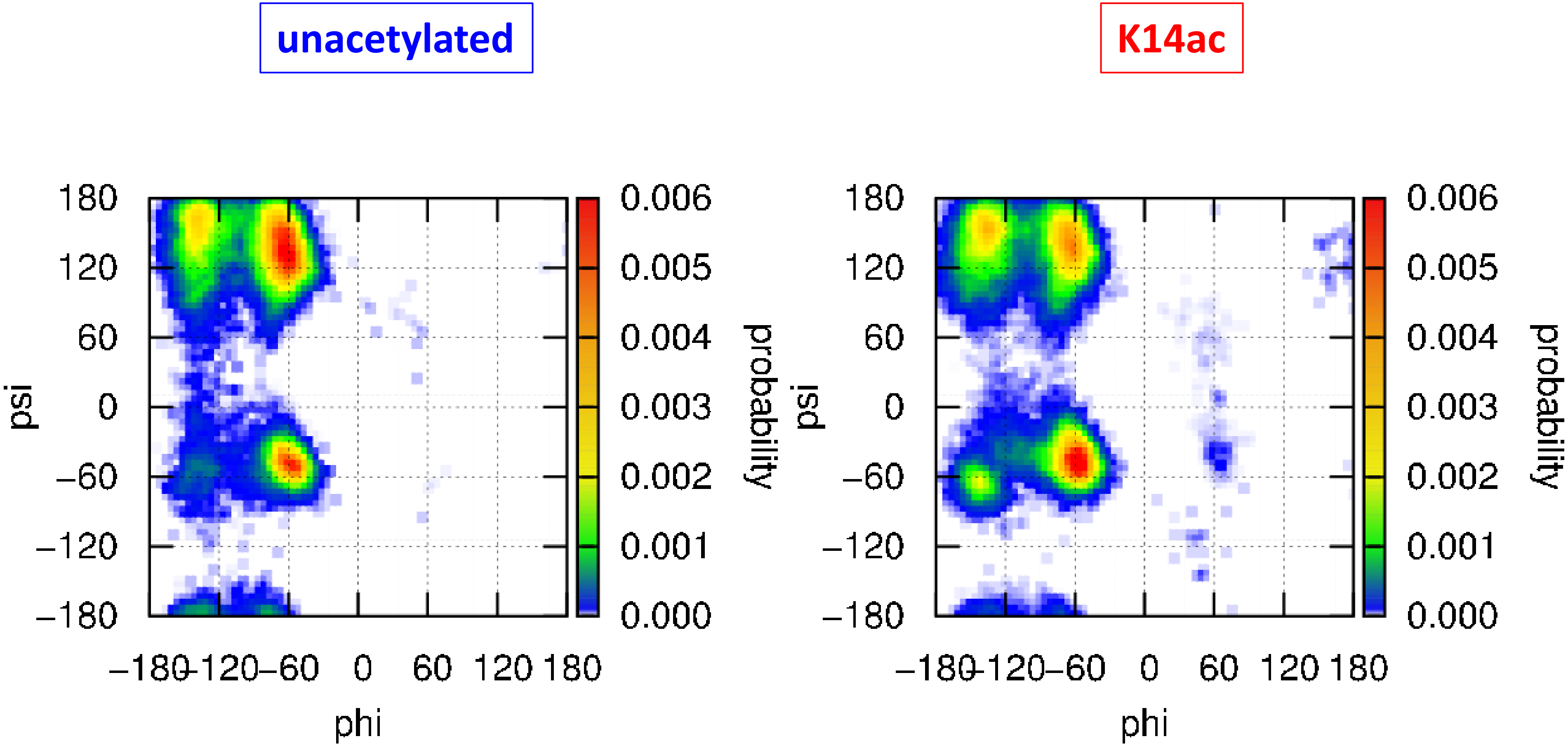
unacetylated
K14ac
180
120
60
0
−60
−120
−180
psi
−180 −120 −60 0 60 120 180
phi
0.006
0.005
0.004
0.003
0.002
0.001
0.000
probability

20 LEU

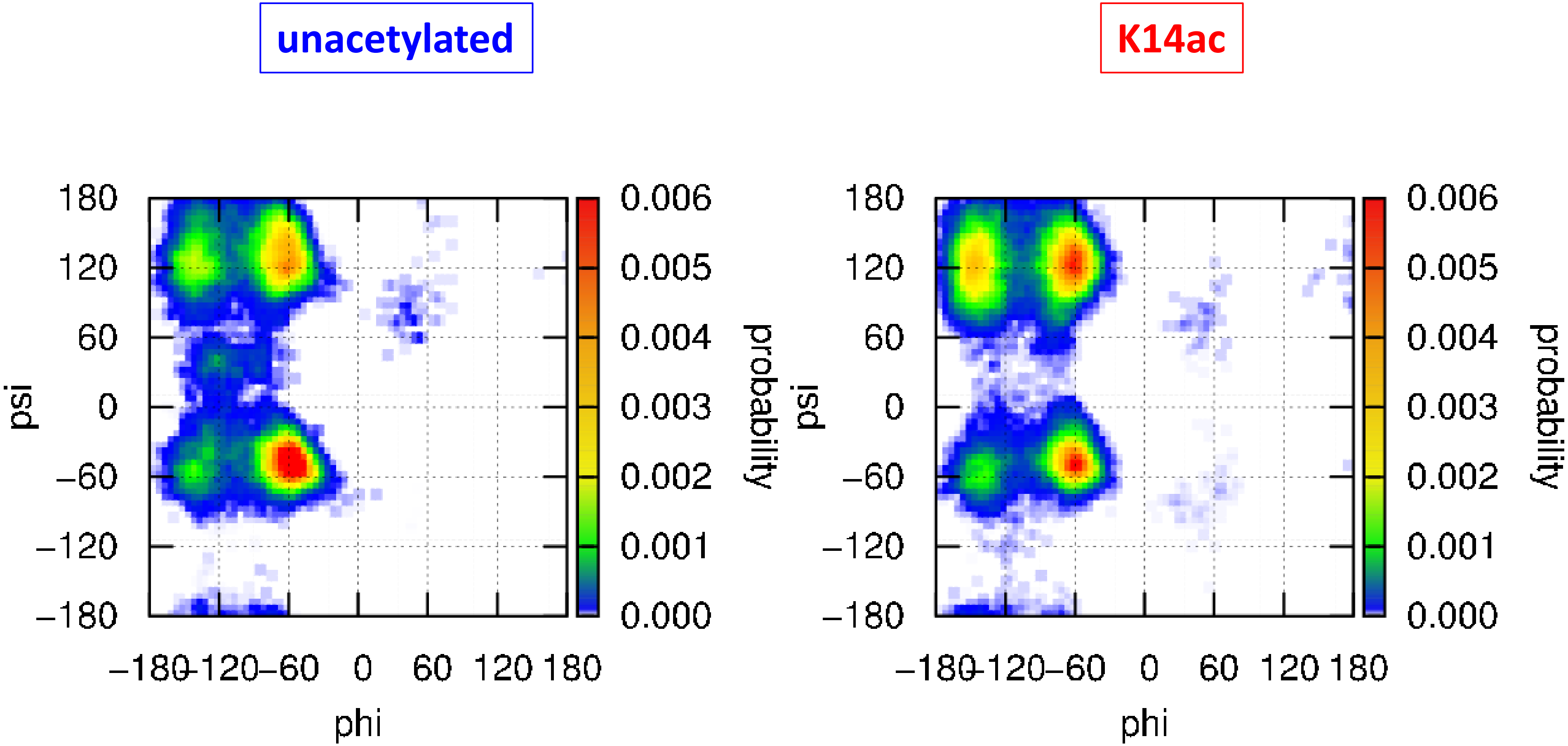
unacetylated
K14ac
psi
phi
probability
180
120
60
0
−60
−120
−180
−180−120−60 0 60 120 180
0.006
0.005
0.004
0.003
0.002
0.001
0.000

21 ALA

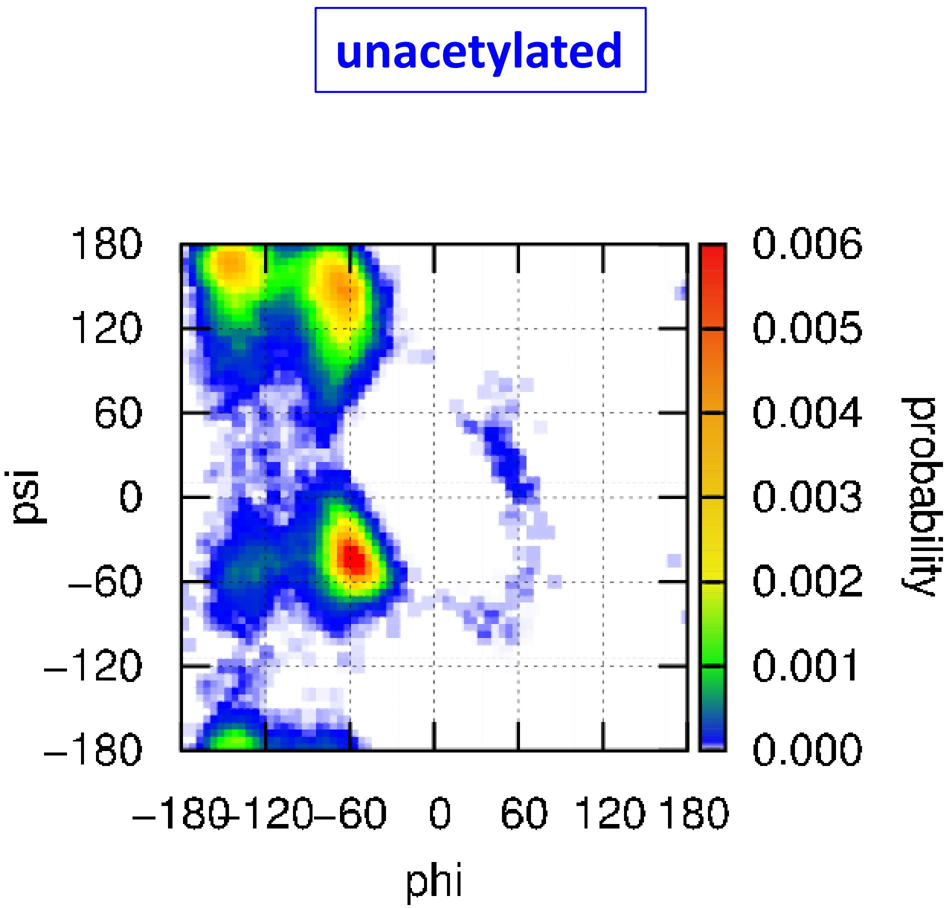

unacetylated
180
120
60
0
−60
−120
−180
psi
−180
−120
−60
0
60
120
180
phi
0.006
0.005
0.004
0.003
0.002
0.001
0.000
probability

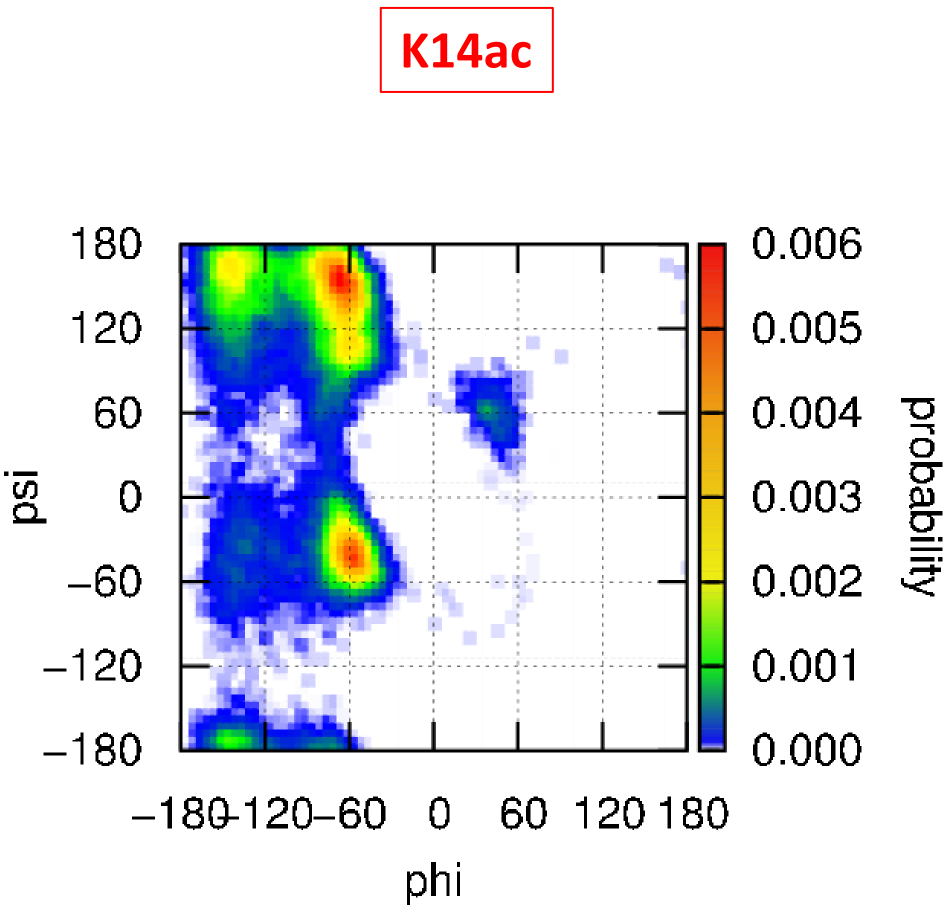

K14ac
180
120
60
0
−60
−120
−180
psi
−180
−120
−60
0
60
120
180
phi
0.006
0.005
0.004
0.003
0.002
0.001
0.000
probability

22 THR

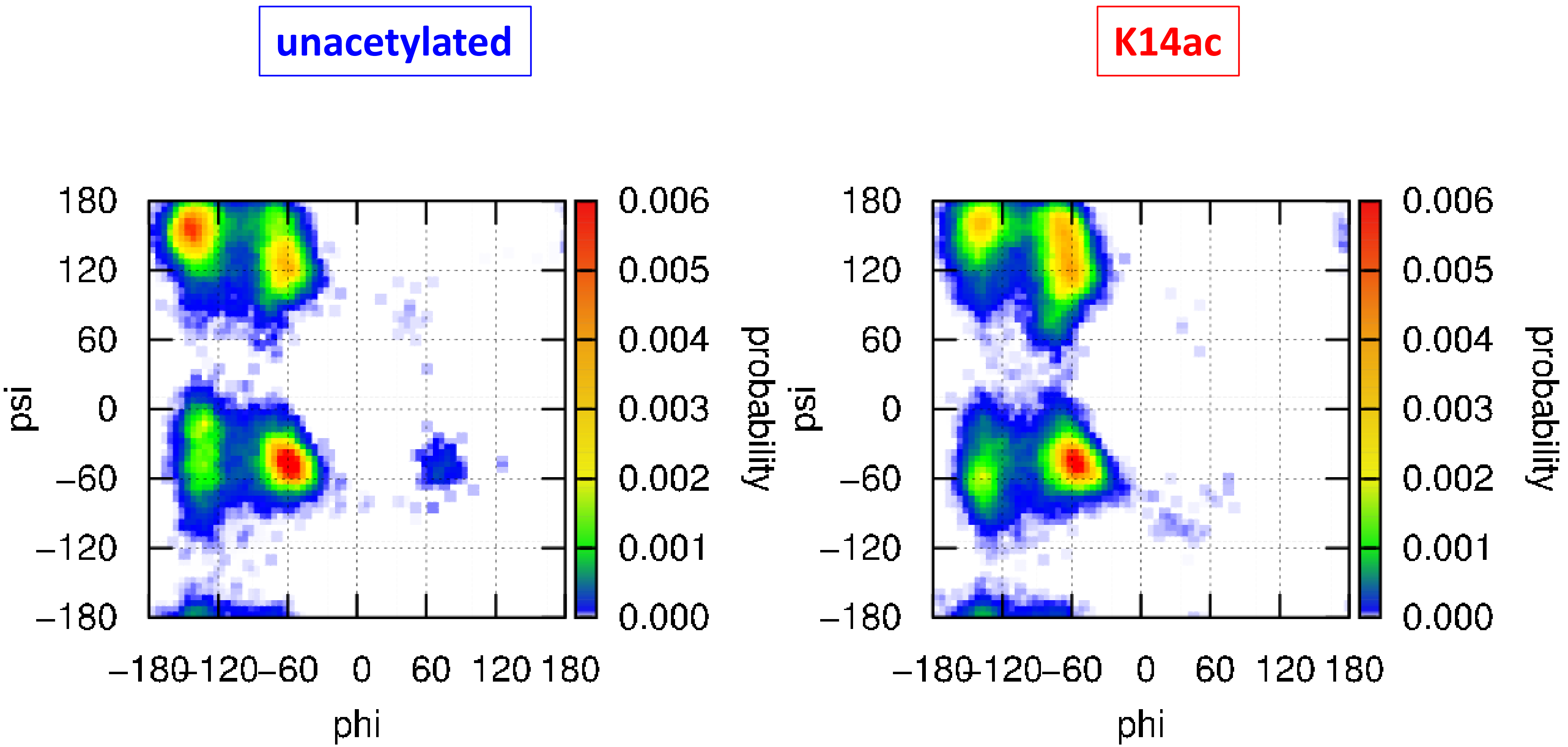
unacetylated
K14ac
180
120
60
0
−60
−120
−180
psi
−180 −120 −60 0 60 120 180
phi
0.006
0.005
0.004
0.003
0.002
0.001
0.000
probability

23 LYS+

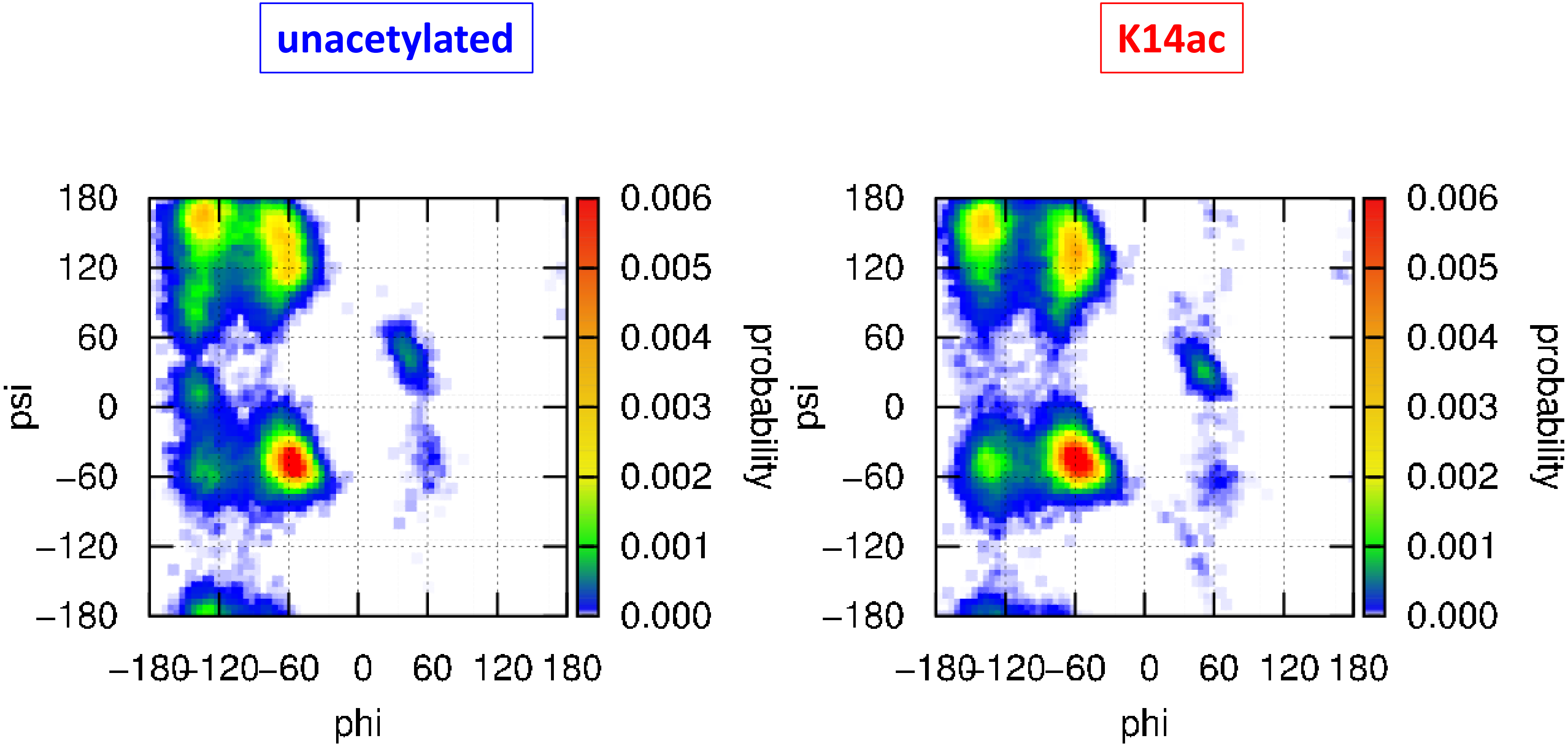
unacetylated
K14ac
180
120
60
0
−60
−120
−180
psi
−180−120−60 0 60 120 180
phi
0.006
0.005
0.004
0.003
0.002
0.001
0.000
probability

24 ALA

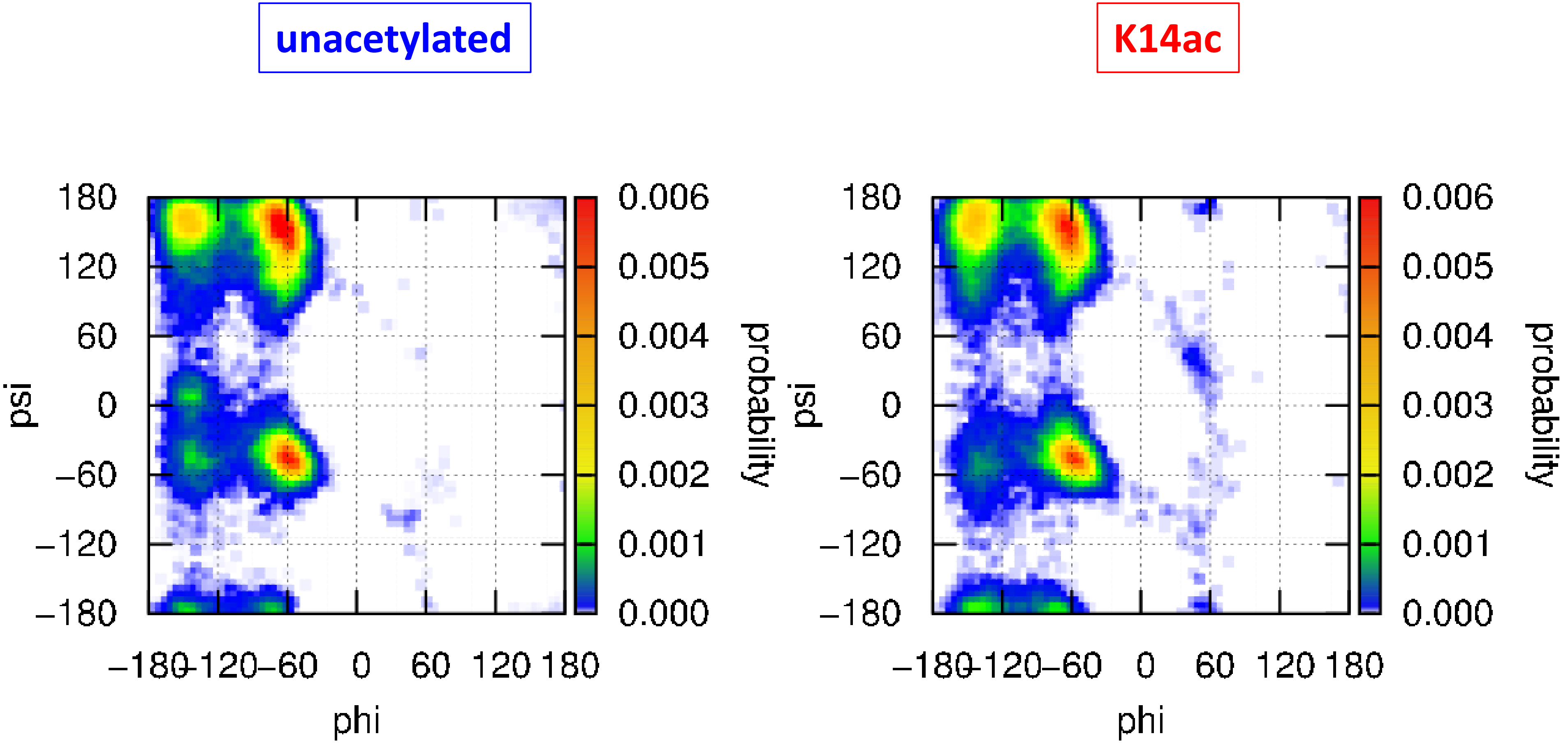

unacetylated
K14ac
180
120
60
0
−60
−120
−180
psi
−180−120−60 0 60 120 180
phi
0.006
0.005
0.004
0.003
0.002
0.001
0.000
probability

25 ALA

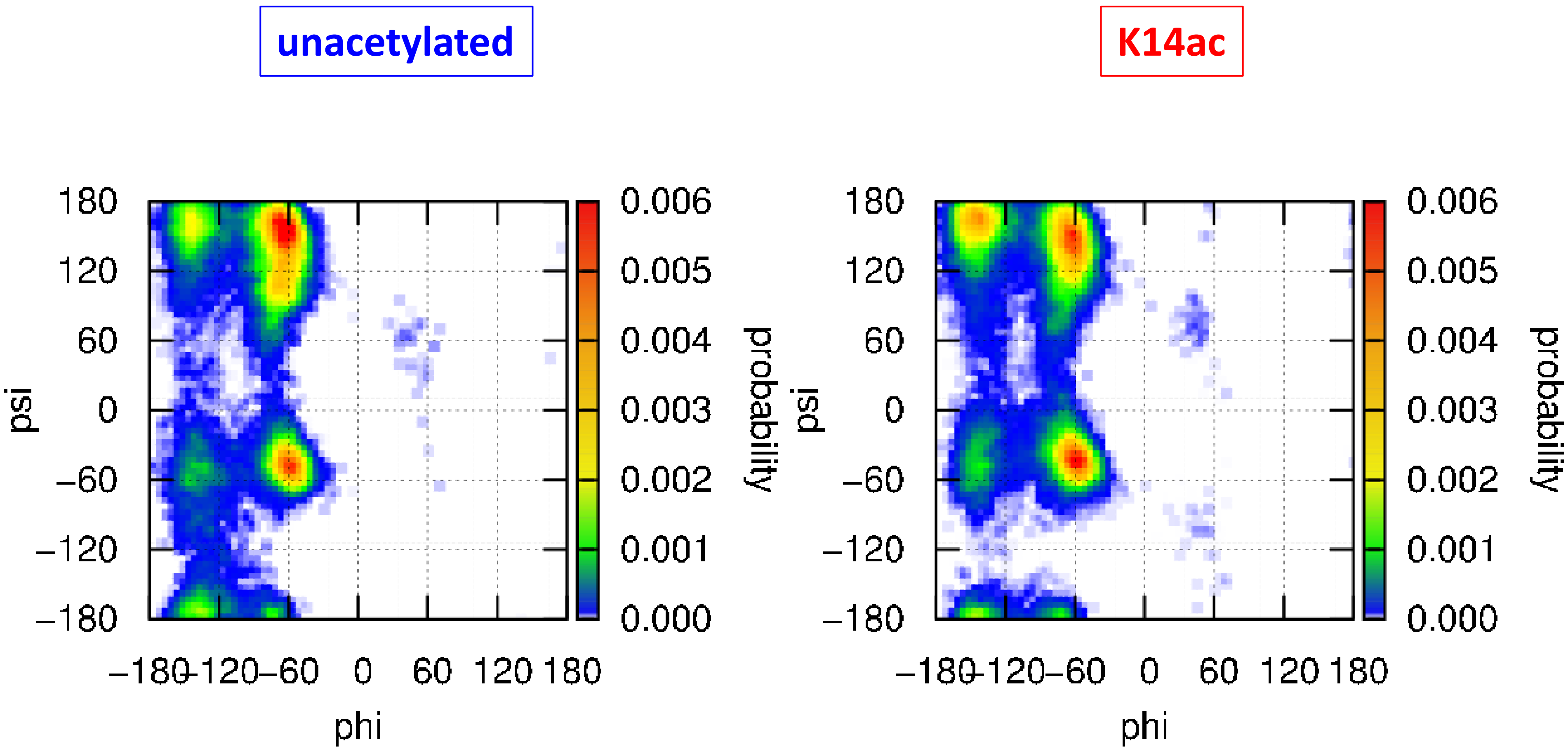
unacetylated
K14ac
psi
phi
probability
180
120
60
0
−60
−120
−180
−180
−120
−60
0
60
120
180
0.006
0.005
0.004
0.003
0.002
0.001
0.000

26 ARG+

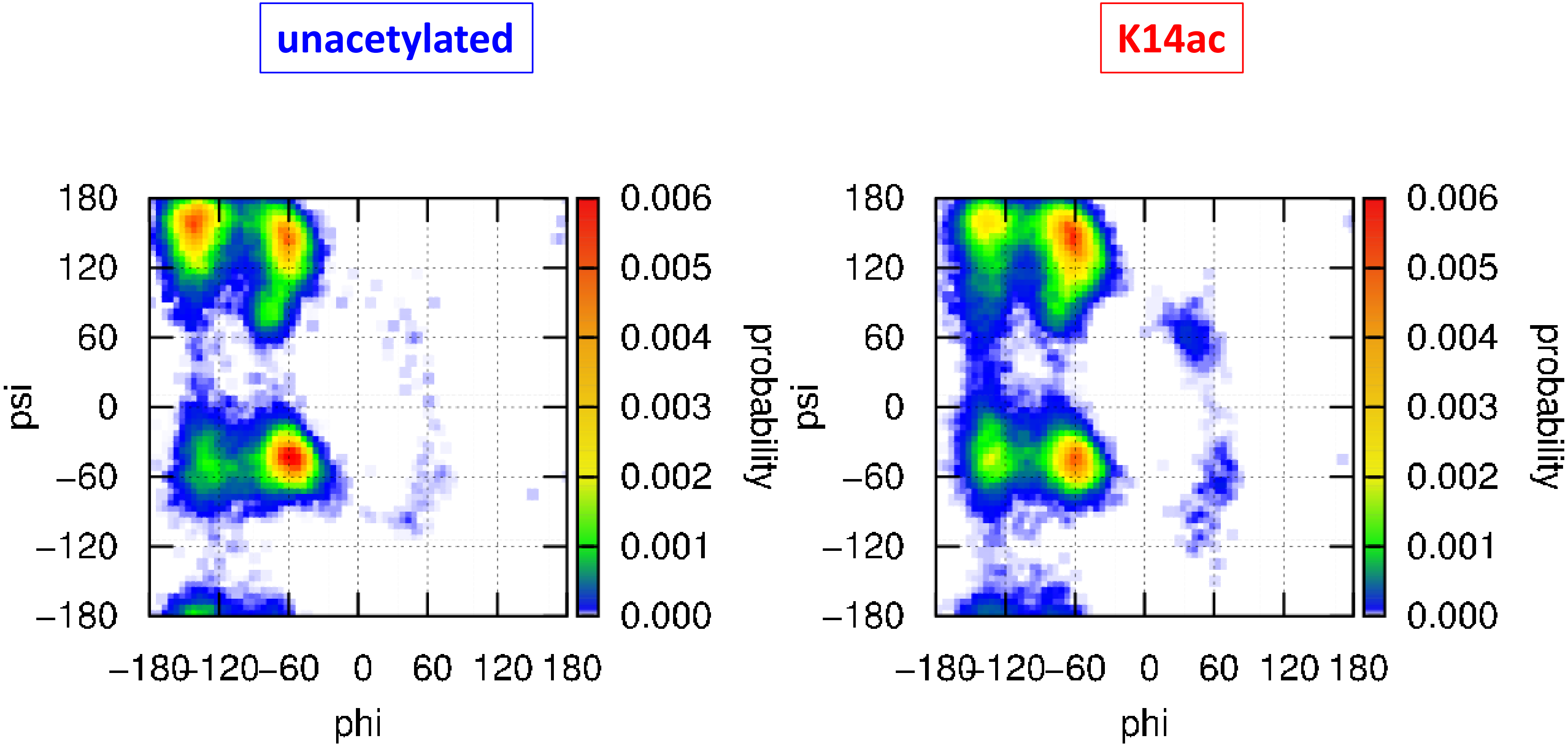
unacetylated
K14ac
180
120
60
0
−60
−120
−180
psi
−180
−120
−60
0
60
120
180
phi
0.006
0.005
0.004
0.003
0.002
0.001
0.000
probability

27 LYS+

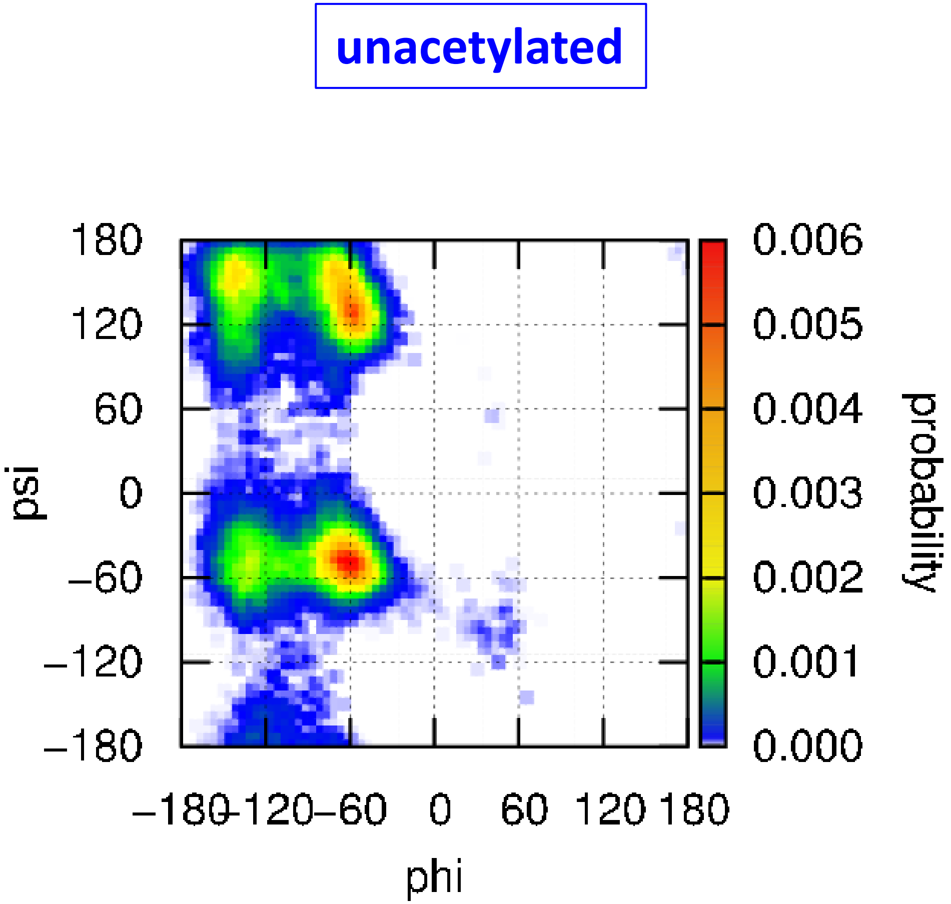

unacetylated
180
120
60
0
−60
−120
−180
psi
−180−120−60 0 60 120 180
phi
0.006
0.005
0.004
0.003
0.002
0.001
0.000
probability

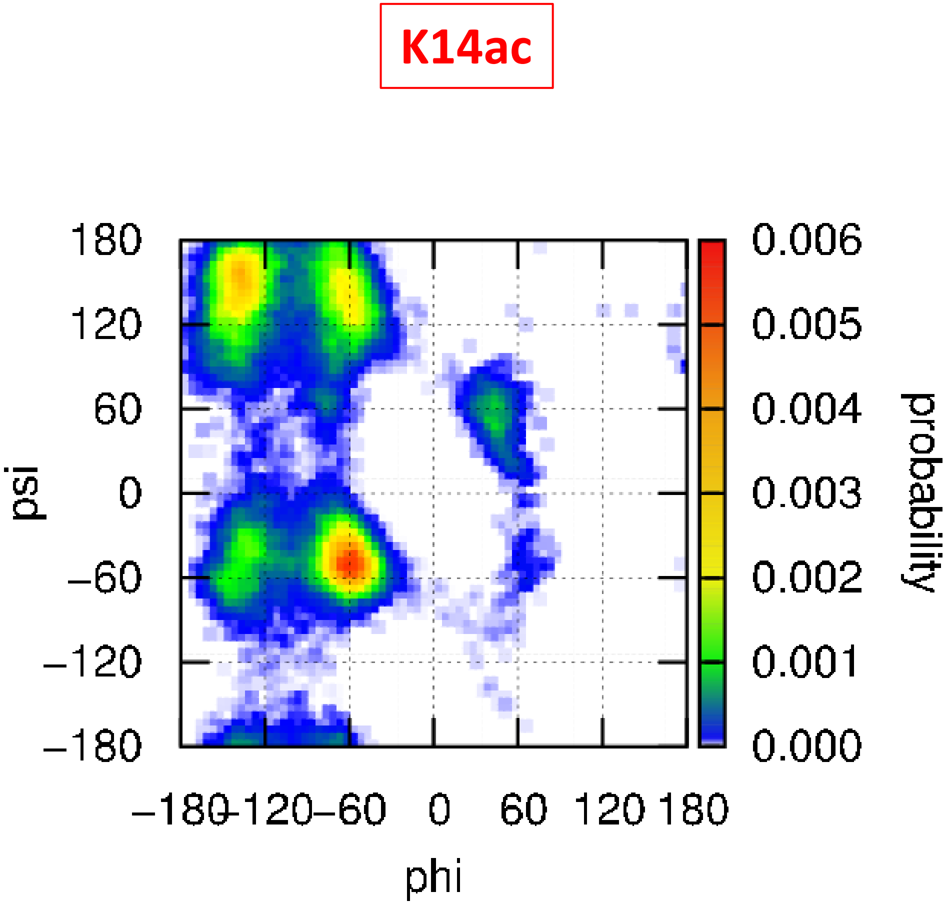

K14ac
180
120
60
0
−60
−120
−180
psi
−180−120−60 0 60 120 180
phi
0.006
0.005
0.004
0.003
0.002
0.001
0.000
probability

28 SER

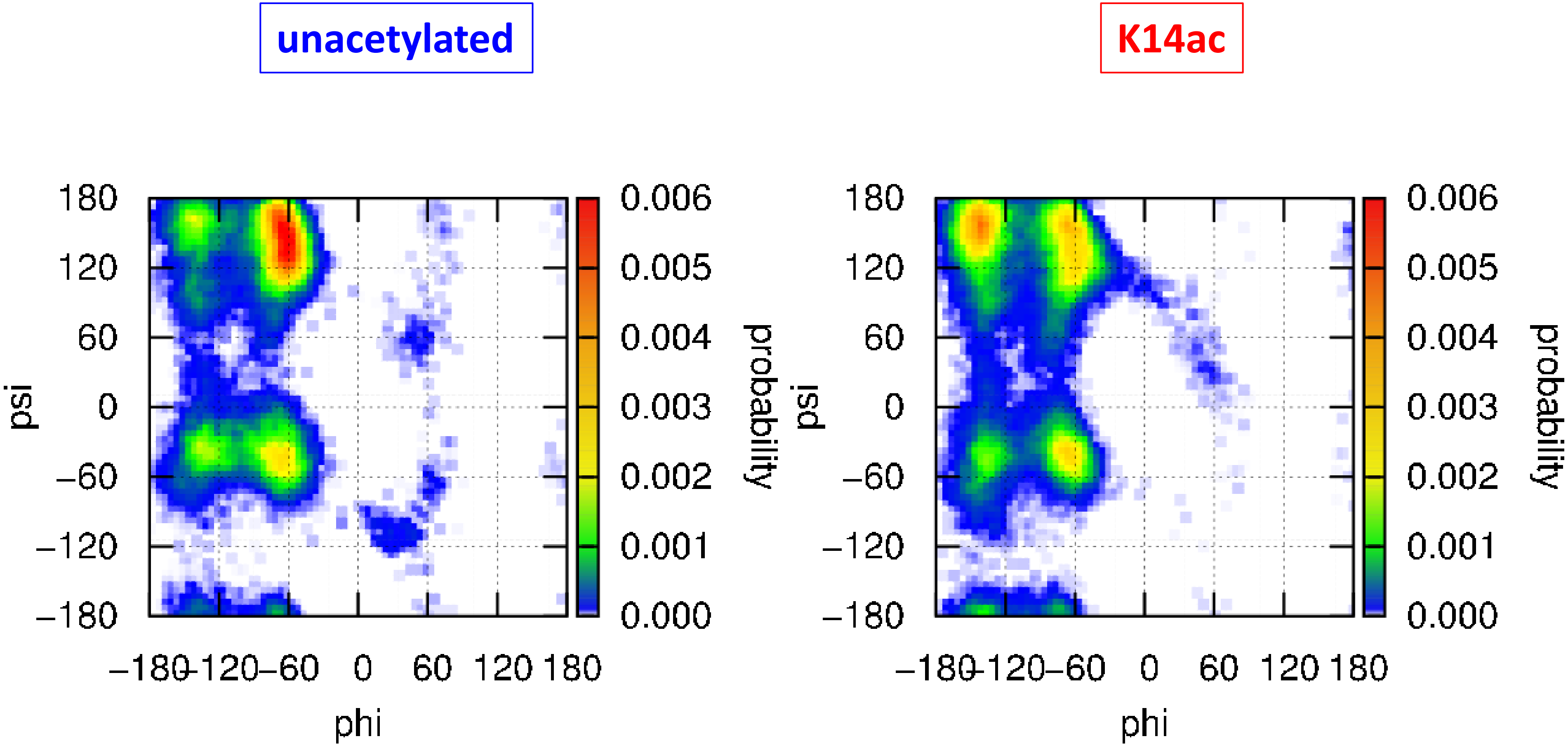
unacetylated
K14ac
180
120
60
0
−60
−120
−180
psi
−180−120−60 0 60 120 180
phi
0.006
0.005
0.004
0.003
0.002
0.001
0.000
probability

29 ALA

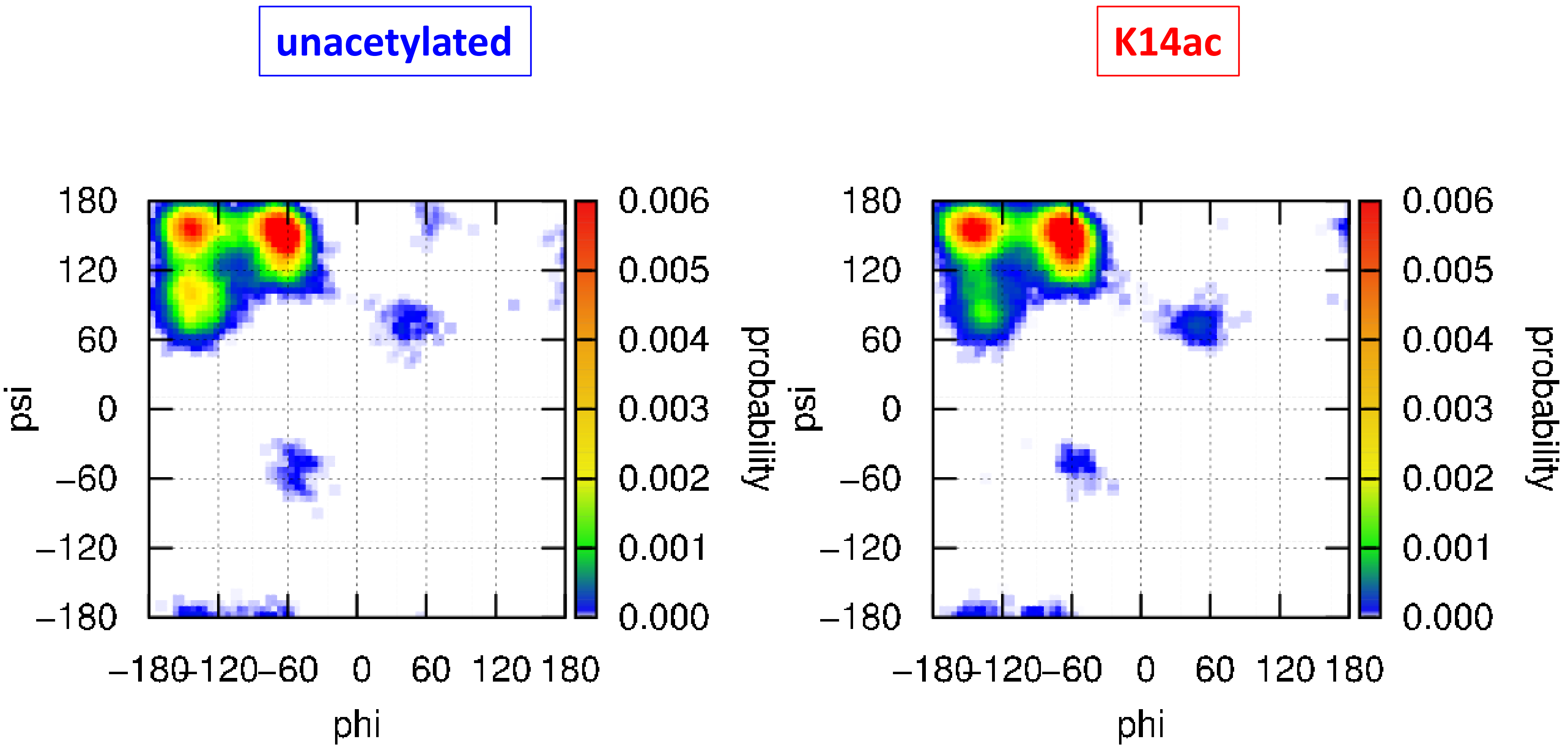
unacetylated
K14ac
psi
phi
probability
180
120
60
0
−60
−120
−180
−180−120−60 0 60 120 180
0.006
0.005
0.004
0.003
0.002
0.001
0.000

30 PRO

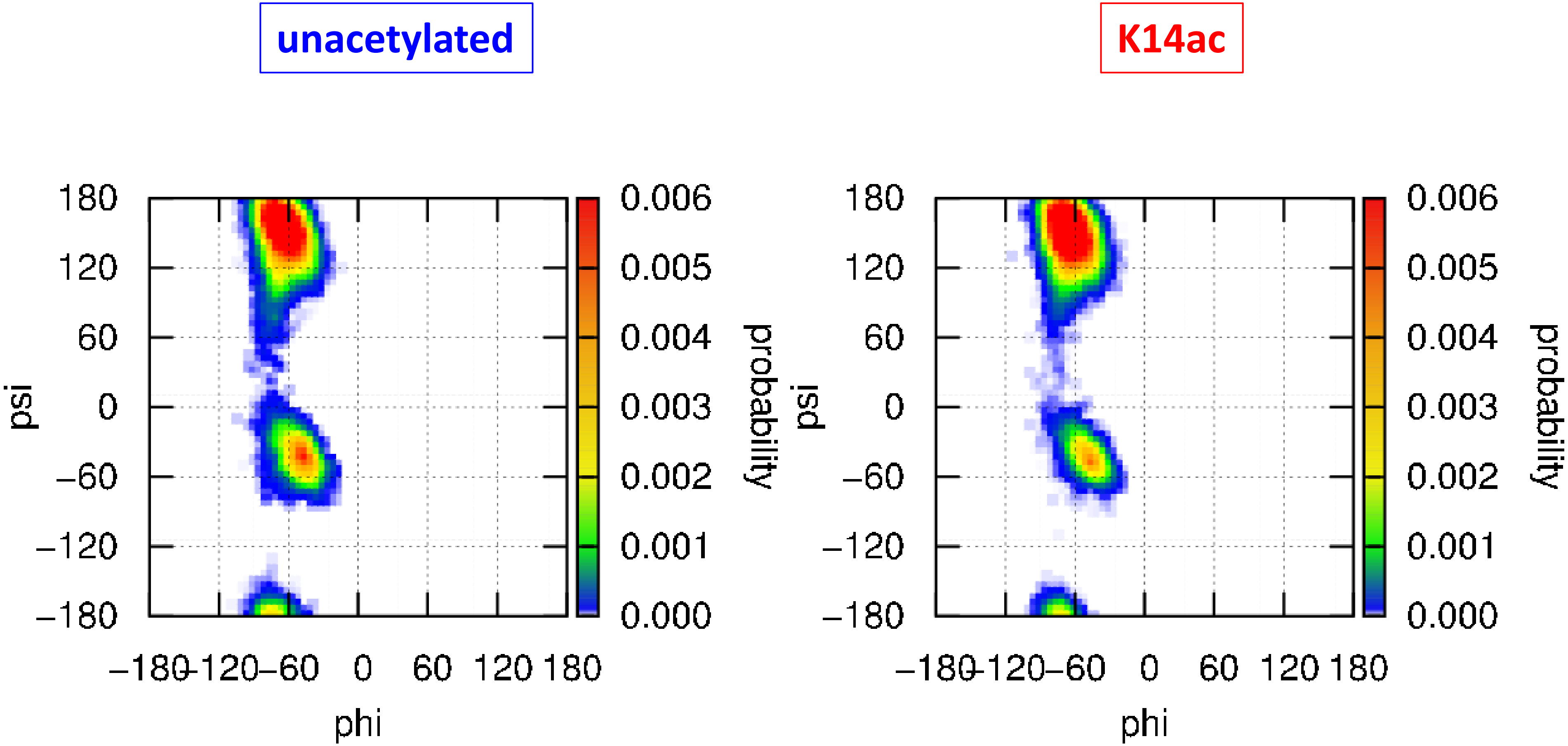
unacetylated
K14ac
psi
phi
probability
180
120
60
0
−60
−120
−180
−180
−120
−60
0
60
120
180
0.006
0.005
0.004
0.003
0.002
0.001
0.000

31 ALA

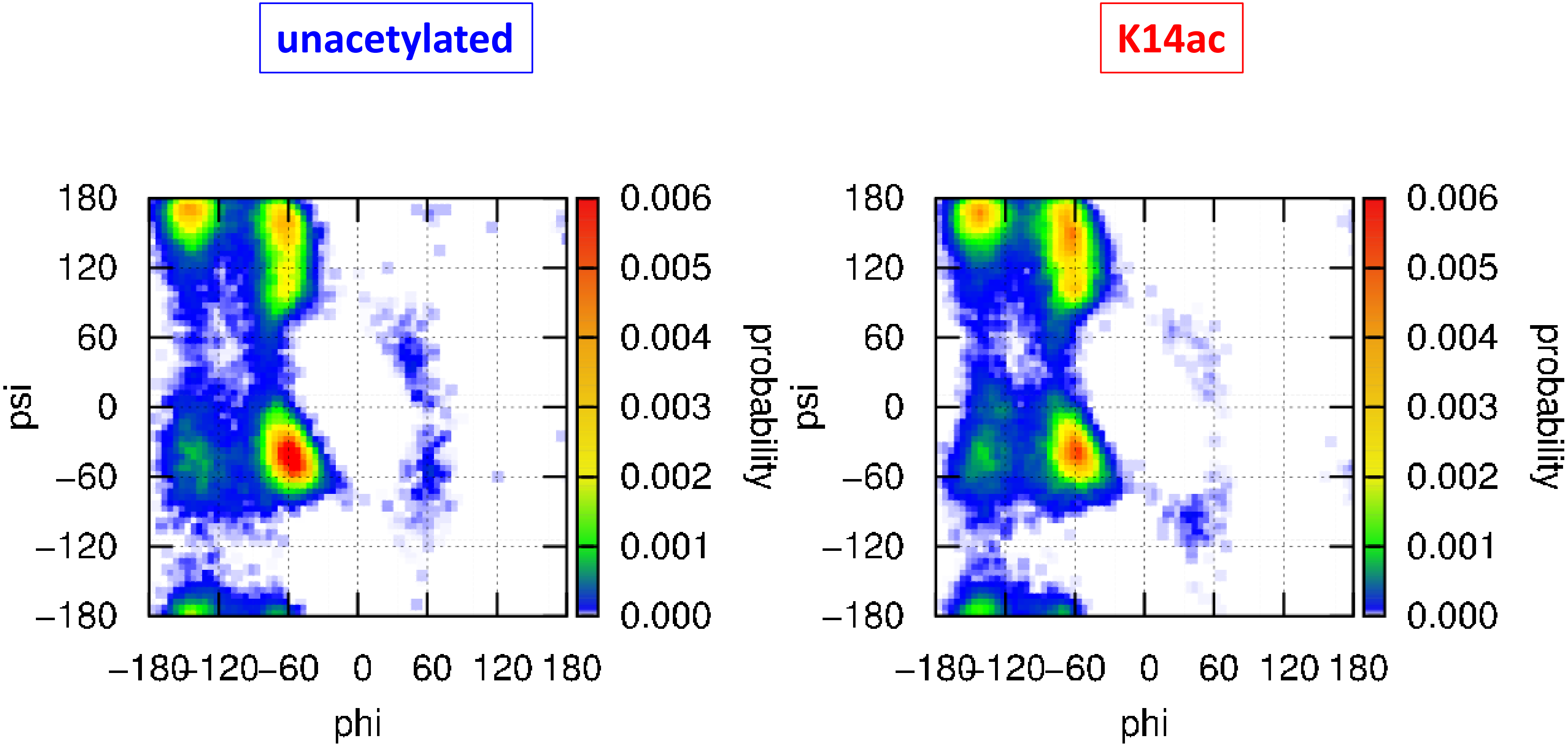

unacetylated
K14ac
180
120
60
0
−60
−120
−180
psi
−180−120−60 0 60 120 180
phi
0.006
0.005
0.004
0.003
0.002
0.001
0.000
probability

32 THR

unacetylated **K14ac**

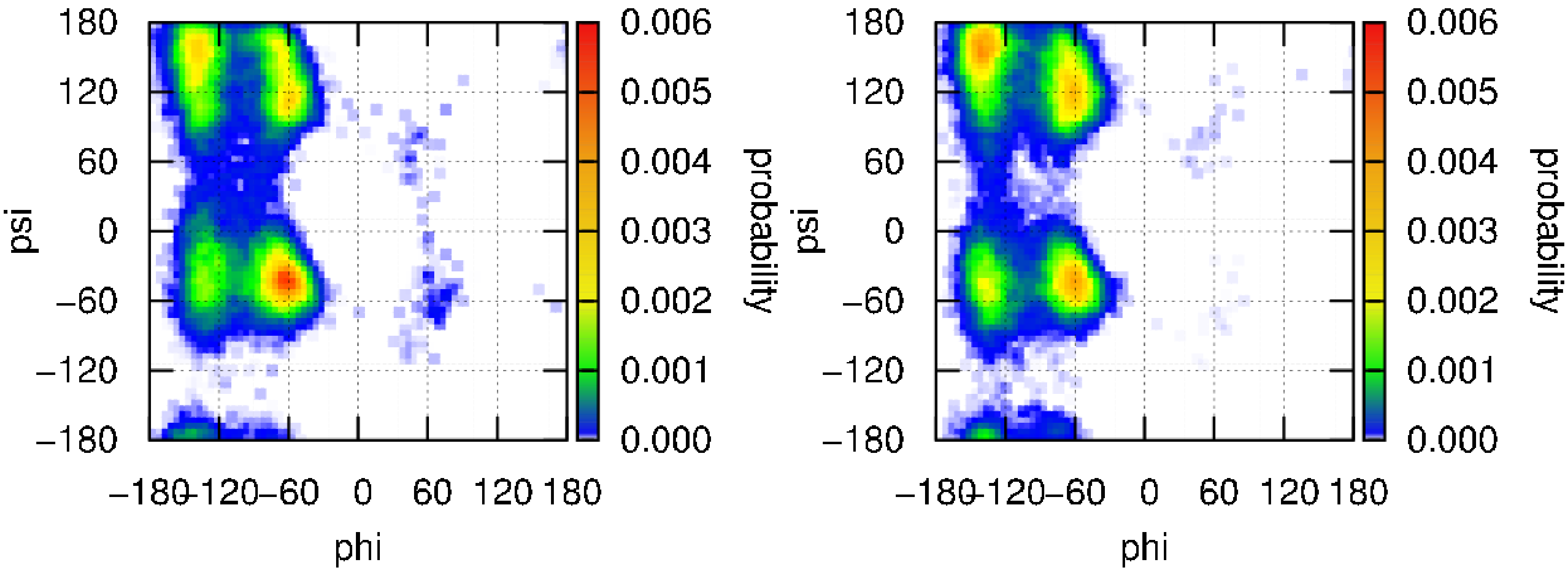

33 GLY

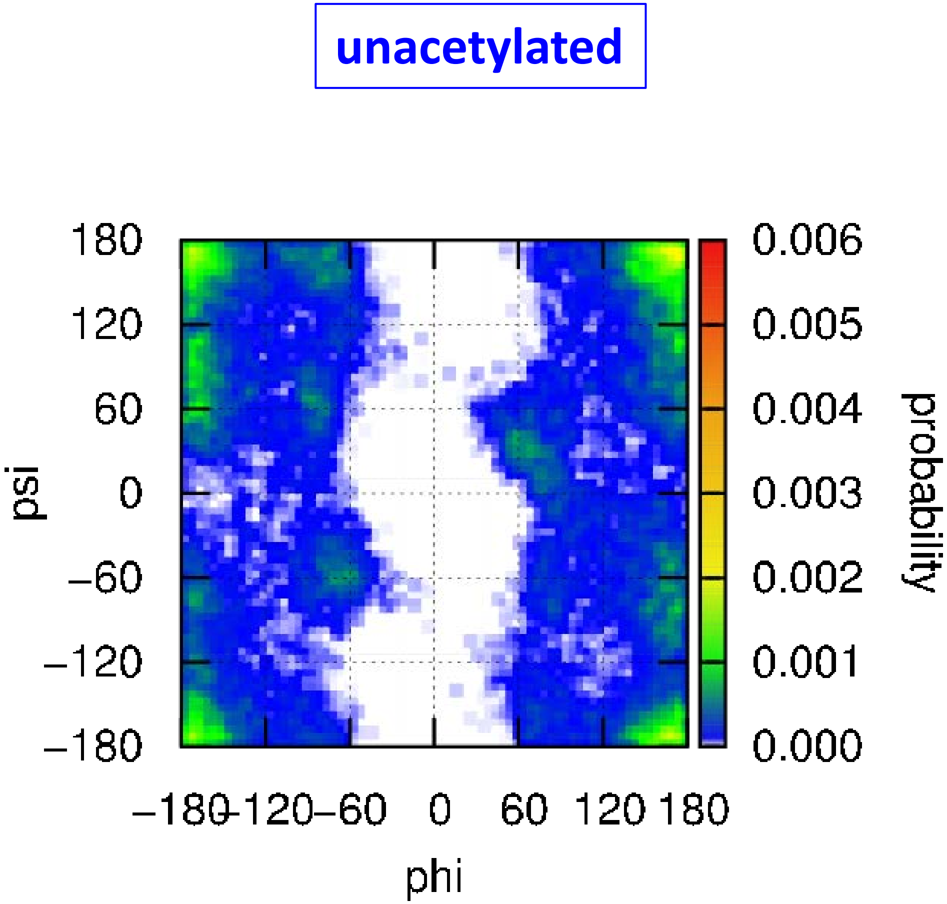
unacetylated
180
120
60
0
−60
−120
−180
psi
−180 −120 −60 0 60 120 180
phi
0.006
0.005
0.004
0.003
0.002
0.001
0.000
probability

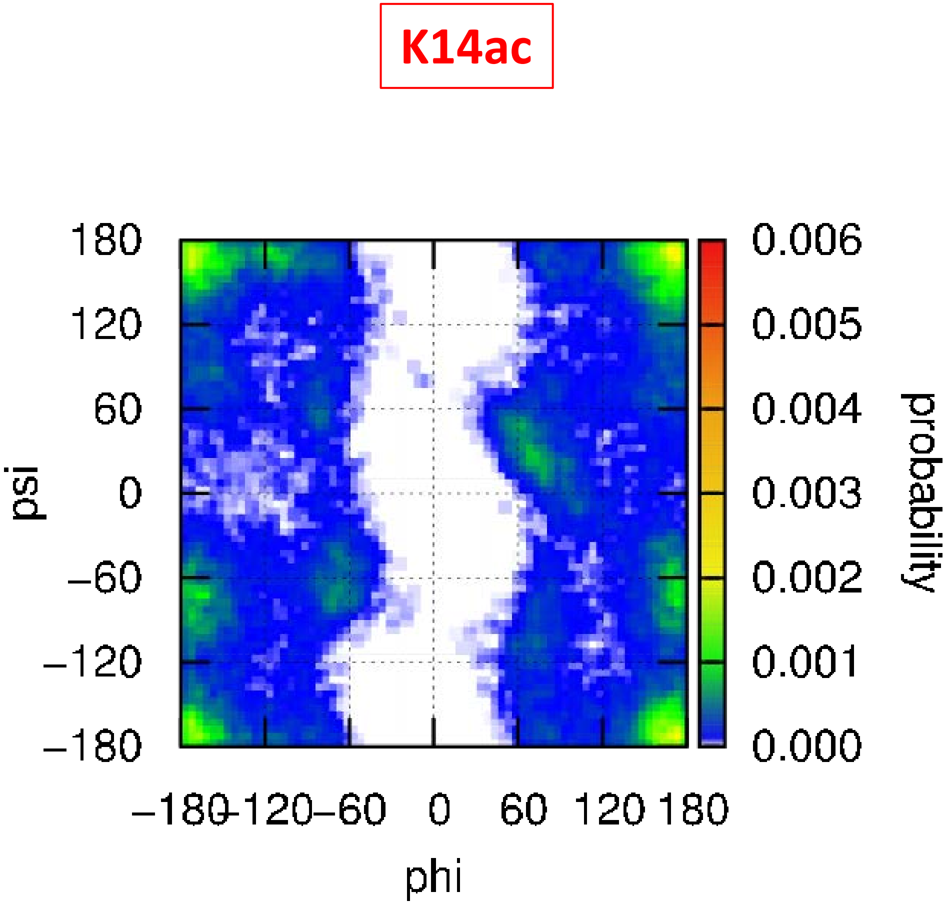
K14ac
180
120
60
0
−60
−120
−180
psi
−180 −120 −60 0 60 120 180
phi
0.006
0.005
0.004
0.003
0.002
0.001
0.000
probability

34 GLY

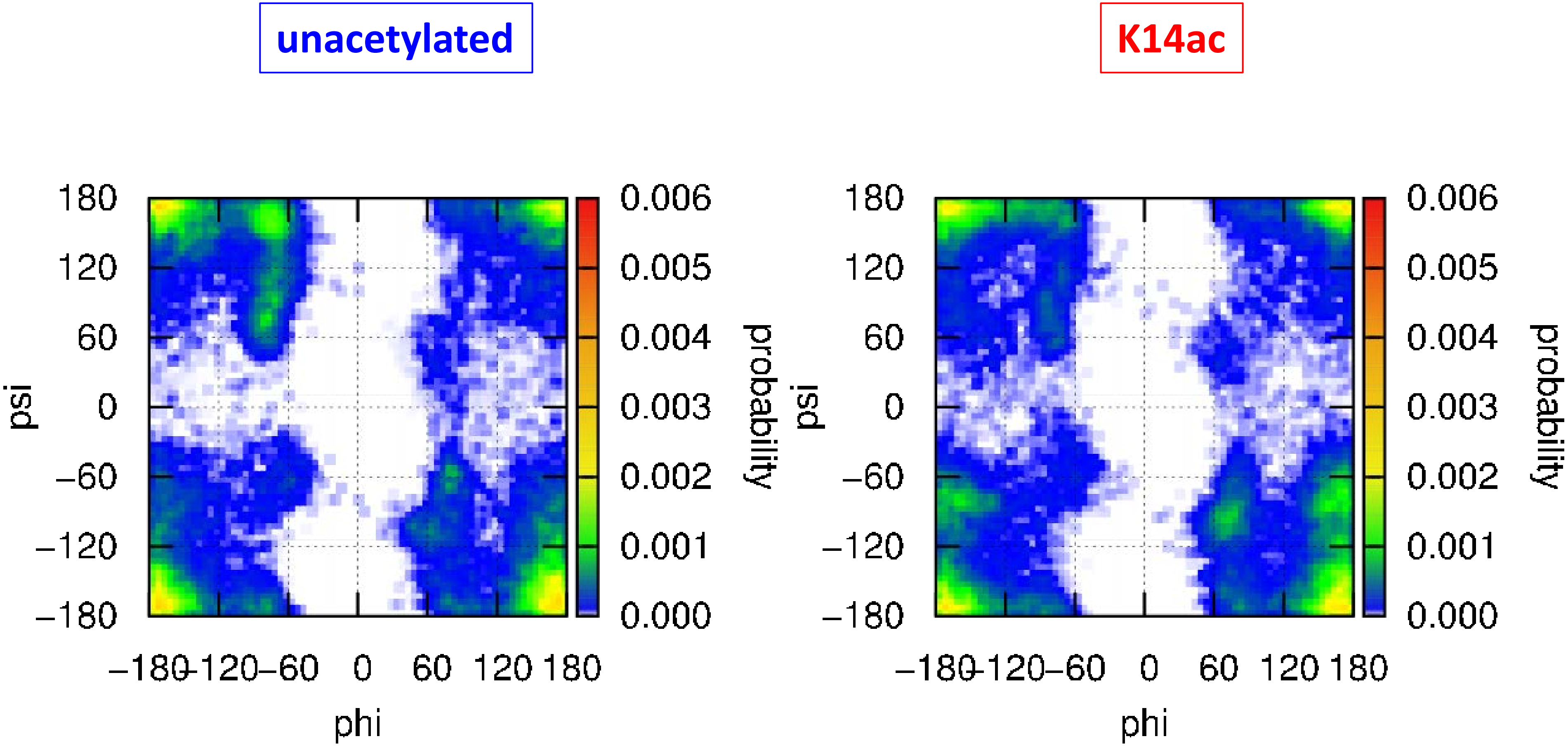

unacetylated
K14ac
psi
phi
probability
180
120
60
0
−60
−120
−180
0.006
0.005
0.004
0.003
0.002
0.001
0.000

35 VAL

unacetylated

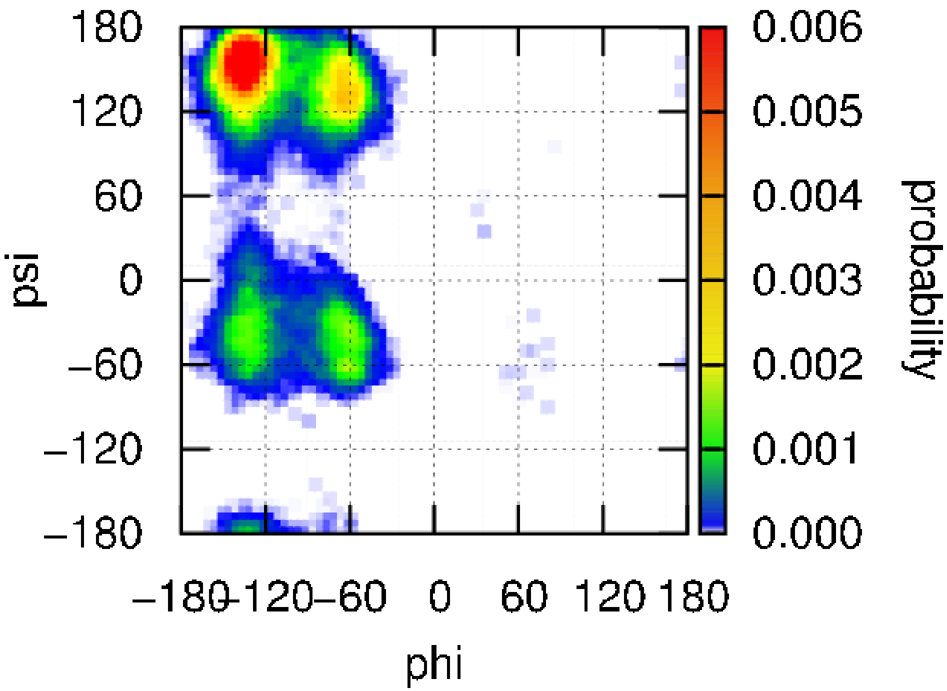

K14ac

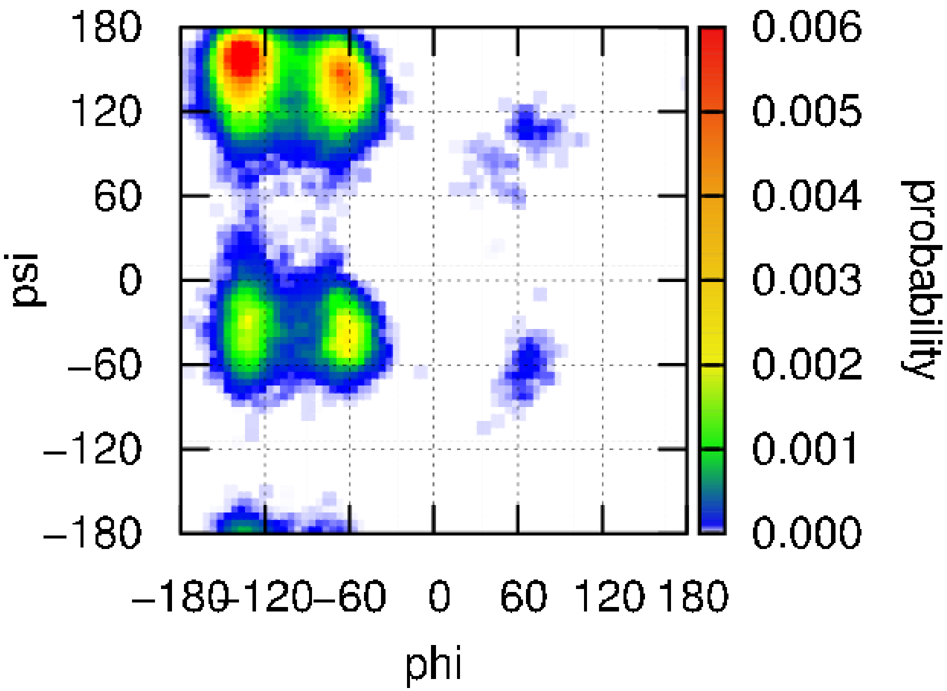

36 LYS+

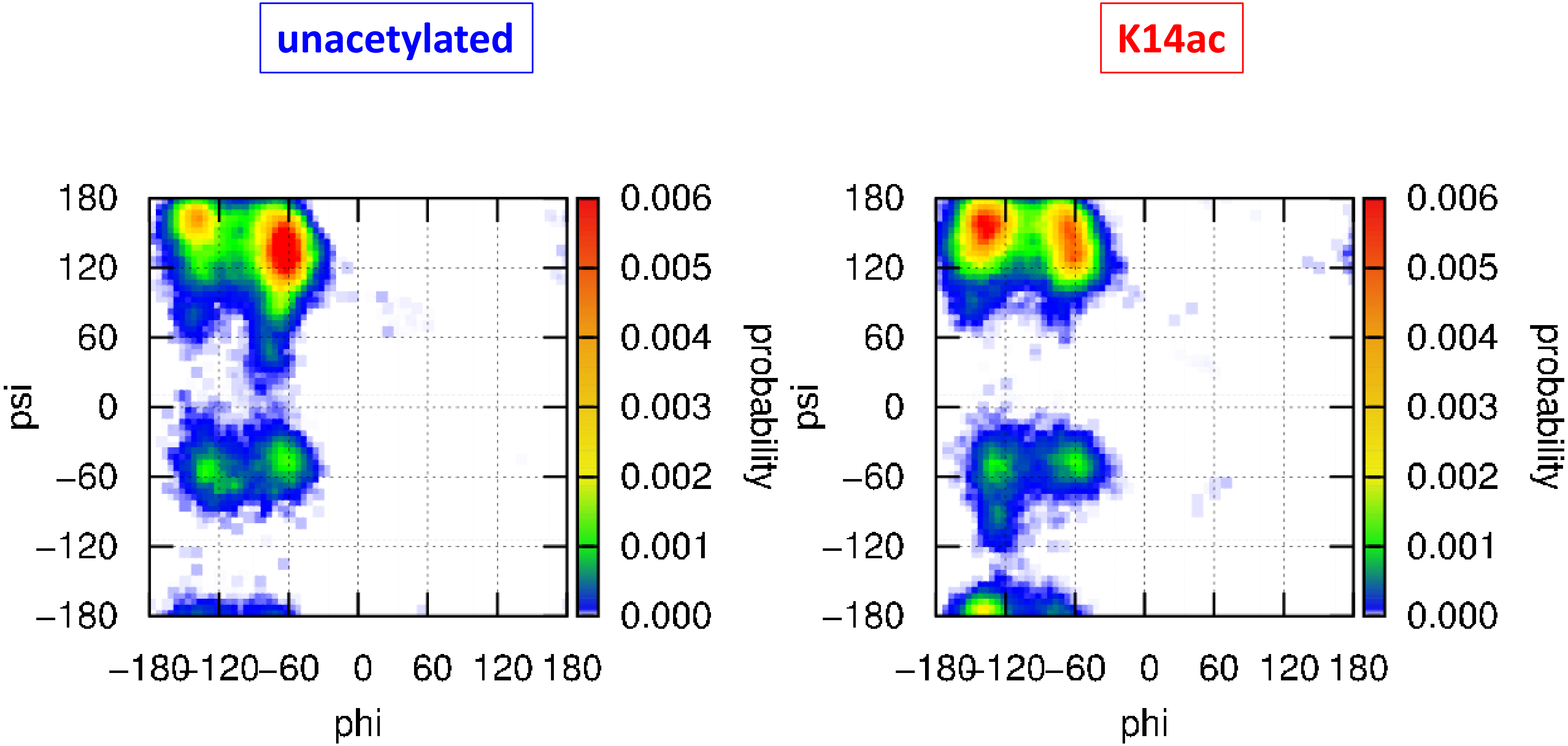
unacetylated
K14ac
psi
phi
probability
180
120
60
0
−60
−120
−180
−180−120−60 0 60 120 180
0.006
0.005
0.004
0.003
0.002
0.001
0.000

37 LYS+

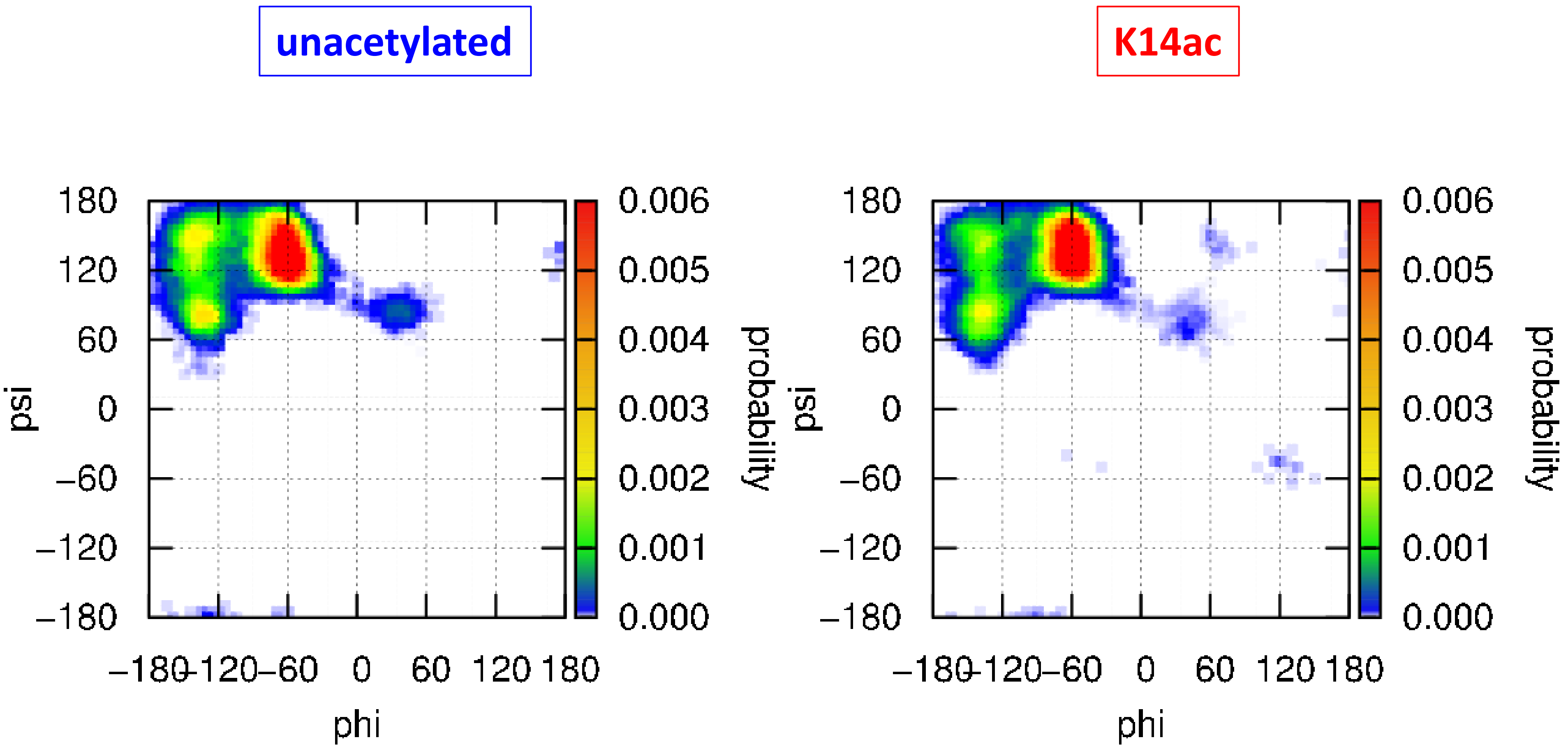

unacetylated
K14ac
180
120
60
0
−60
−120
−180
psi
−180 −120 −60 0 60 120 180
phi
0.006
0.005
0.004
0.003
0.002
0.001
0.000
probability

38 PRO

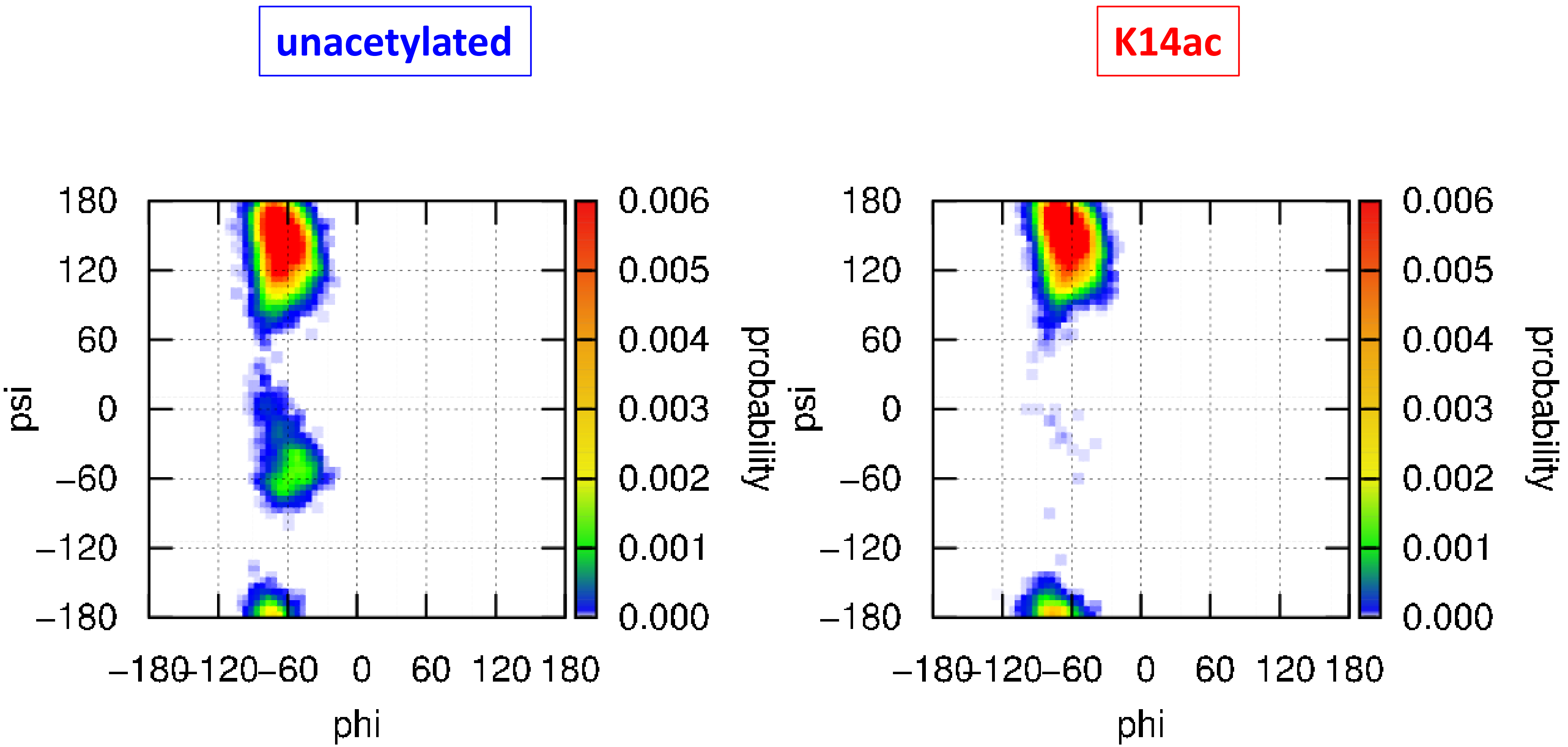
unacetylated
K14ac
180
120
60
0
-60
-120
-180
psi
-180
-120
-60
0
60
120
180
phi
0.006
0.005
0.004
0.003
0.002
0.001
0.000
probability

39 HIS

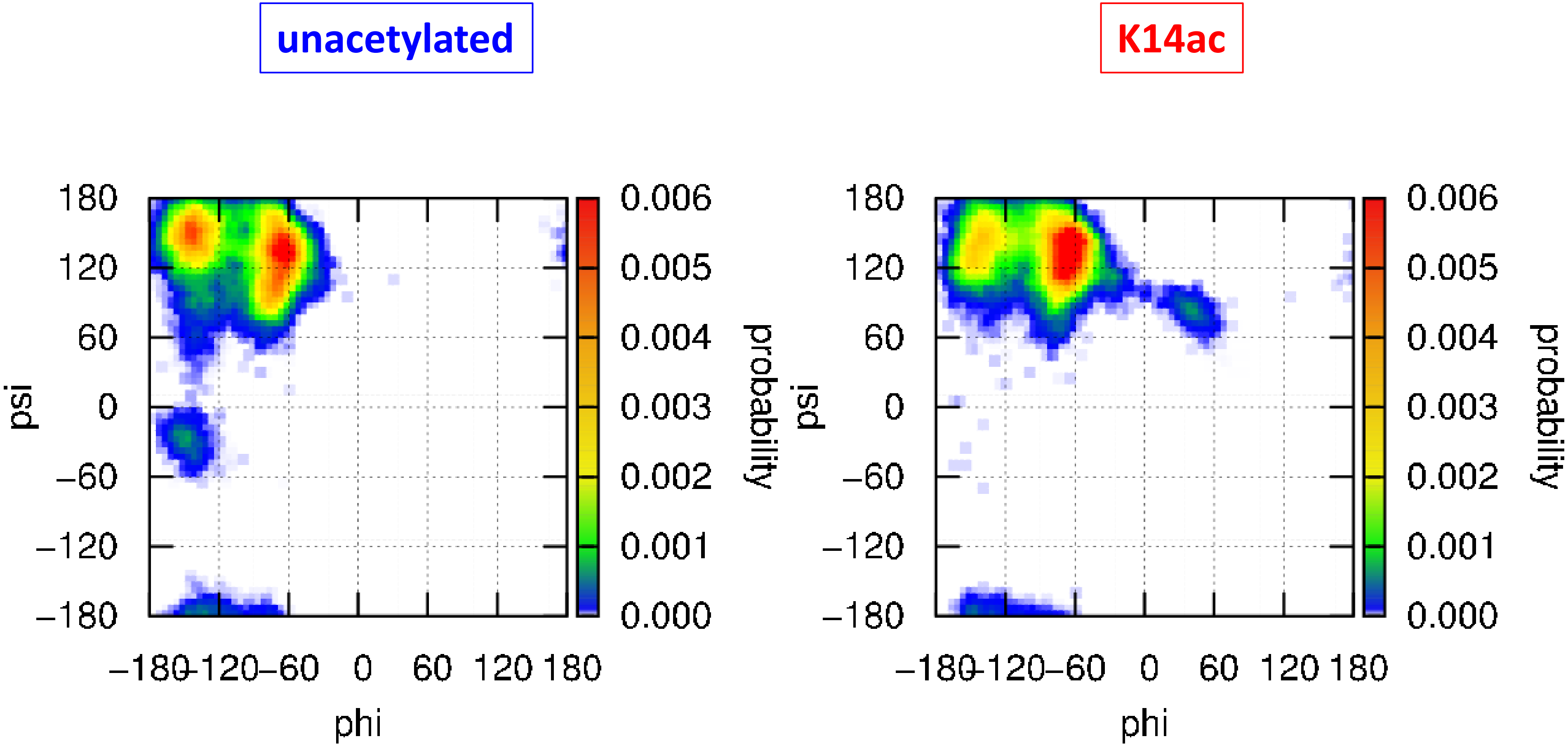
unacetylated
K14ac
180
120
60
0
−60
−120
−180
psi
−180−120−60 0 60 120 180
phi
0.006
0.005
0.004
0.003
0.002
0.001
0.000
probability

40 ARG+

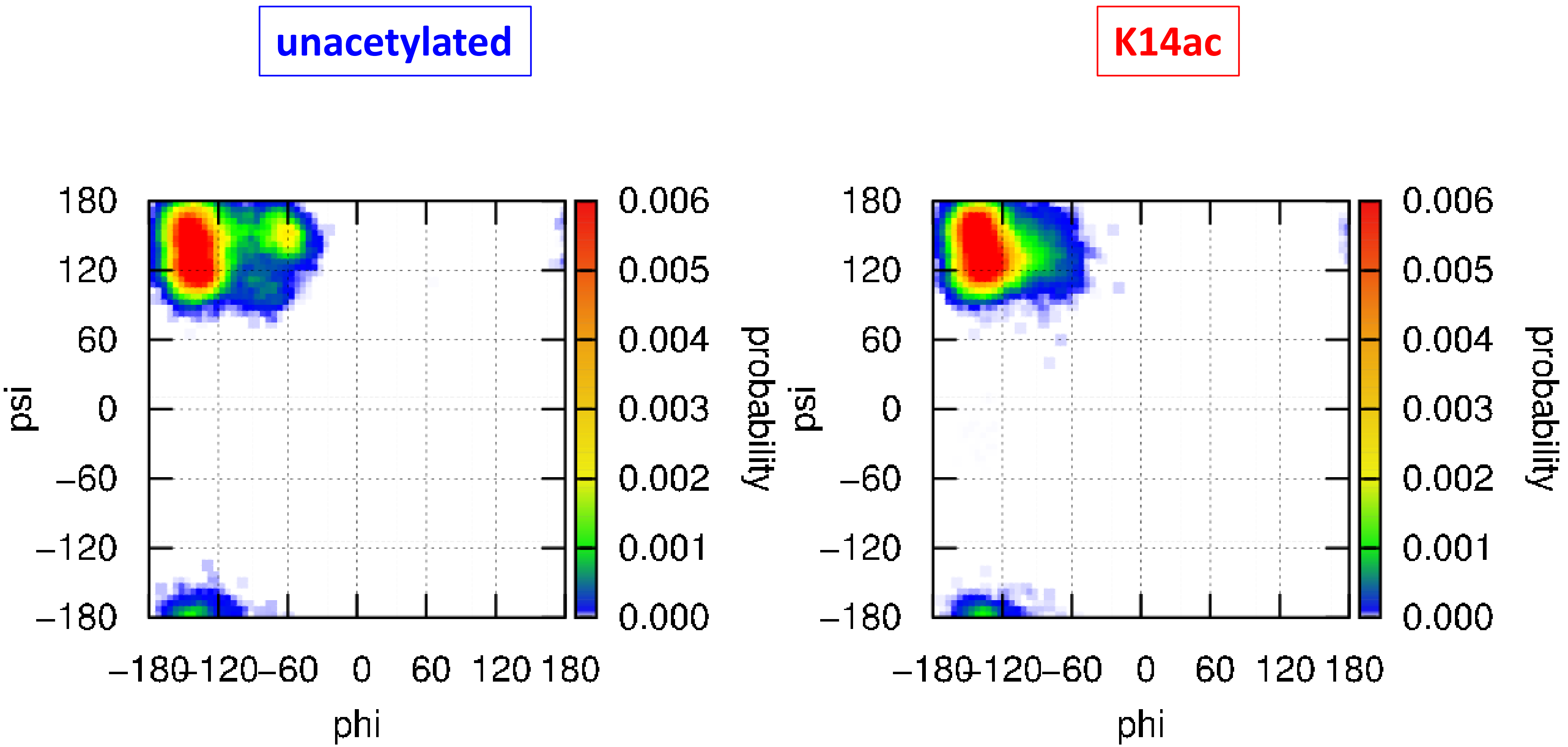

unacetylated
K14ac
180
120
60
0
−60
−120
−180
psi
−180
−120
−60
0
60
120
180
phi
0.006
0.005
0.004
0.003
0.002
0.001
0.000
probability
